# Structural characterization and protective effects of polysaccharides isolated from *Berberis dasystachya* M. fruits on H_2_O_2_-induced oxidative damage in RINm5F pancreatic β-cells

**DOI:** 10.3389/fnut.2025.1643051

**Published:** 2025-08-06

**Authors:** Xiaohang Lu, Lijuan Han, Yongrong Deng

**Affiliations:** ^1^State Key Laboratory of Plateau Ecology and Agriculture, Qinghai University, Xining, China; ^2^College of Agriculture and Animal Husbandry, Qinghai University, Xining, China

**Keywords:** *Berberis dasystachya* M., metabolomics, oxidative stress, pancreatic β-cells, polysaccharides, structural characterization

## Abstract

Our previous research demonstrated the hypoglycemic and antioxidant properties of crude polysaccharides from *Berberis dasystachya* M. (BDP), but the structural characteristics and underlying mechanisms were unclear. In this study, a homogeneous polysaccharide, BDP-I, was isolated and structurally characterized. BDP-I had a molecular weight of 3.947 kDa and consisted of arabinose, galactose, glucose, and galacturonic acid, mainly linked through →6)-β-D-GalAp-(1→ glycosidic bonds.An oxidative stress model was established by treating RIN-m5F pancreatic islet cells with 250 μmol/L H_2_O_2_ for 3 hours. BDP-I intervention at concentrations ranging from 0.0625 to 0.5 mg/mL significantly improved cell viability, reduced reactive oxygen species (ROS) and thiobarbituric acid reactive substances (TBARS), and enhanced superoxide dismutase (SOD) and catalase (CAT) activities in a dose-dependent manner.Untargeted metabolomics revealed that BDP-I regulated 46 potential biomarkers, effectively reversing metabolic disturbances caused by oxidative stress. In addition, BDP-I modulated the expression of genes associated with apoptosis and mitochondrial damage, including iNOS and NF-κB.These findings suggest that BDP-I exerts protective effects against oxidative damage in pancreatic islet cells through multiple mechanisms, highlighting its potential as a targeted antioxidant agent for functional food or therapeutic applications.

## Graphical abstract

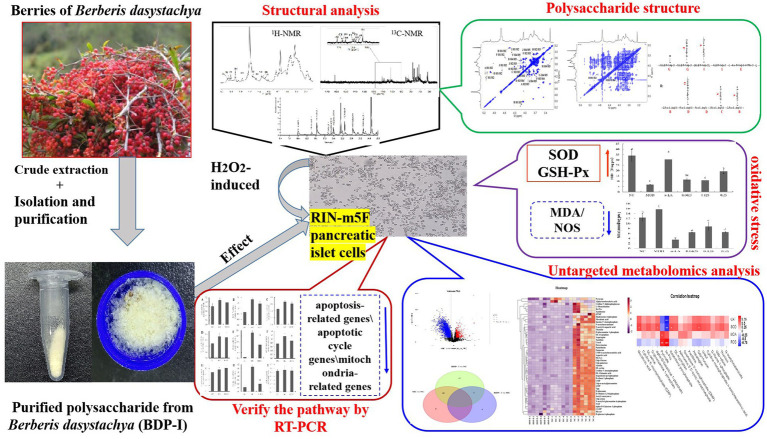

## 1 Introduction

The yellow thorn, also known as *Berberis dasystachya* M., is a homologous berry resource indigenous to the Qinghai-Tibet Plateau (1). The fruit of yellow thorn contains various bioactive constituents, such as phytosterols, organic acids, polysaccharides, and polyphenols (2, 3). Furthermore, the natural and edible fruit polysaccharides derived from this plant exhibits potential for application as a food source, health supplement, or medicinal purposes, especially in preventing and managing diabetes (4). *In vitro*, studies involving BDP exhibited robust anti-oxidative, anti-proliferative, hypoglycemic, and other advantageous biological activities, thereby demonstrating significant potential for development (5). Our research shows that yellow thorn fruit powder and its polysaccharides can lower blood sugar and lipids in diabetic rats, increase insulin levels, and regulate antioxidant enzymes related to ROS metabolism. T2DM is associated with pancreatic β-cell apoptosis and oxidative stress (6). Our previous investigation demonstrated that BDP, within a specific dosage range, could enhance cell survival rates and provide protection against oxidative damage caused by hydrogen peroxide (H_2_O_2_). Nevertheless, the precise mechanism through which BDP intervened in pancreatic β-cells to achieve antioxidative stress effects remained unidentified. Pancreatic beta cells are crucial endocrine cells responsible for the synthesis and secretion of insulin, thus playing a vital role in the regulation of blood glucose levels (7). Studies have demonstrated that oxidative stress can suppress the expression of PDX-1, consequently impacting insulin synthesis and secretion (8). Nevertheless, oxidative stress exerts a multifaceted influence on pancreatic injury, significantly affecting insulin synthesis, secretion, and signaling pathways (9). Currently, extensive research is being conducted on the pathogenesis of oxidative stress-induced diabetes, with particular focus on the mechanisms of oxidative damage through targeted signaling pathways, which has emerged as a prominent area of investigation, the comprehensive examination of oxidative stress pathways is of paramount importance for elucidating the mechanisms of damage and advancing therapeutic strategies for diabetes. Consequently, it is imperative to elucidate the effects and underlying mechanisms of BDP on the antioxidant stress response in pancreatic islet cells, both at the cellular and molecular biology levels.

Plant polysaccharides have garnered considerable attention due to their diverse biological activities, particularly their notable antioxidant properties (10). The antioxidant activity of plant polysaccharides is among the most extensively investigated pharmacological effects. Numerous studies have demonstrated that plant polysaccharides can exert antioxidant effects by modulating signaling pathways, activating enzymes, and scavenging free radicals (11). For instance, plant polysaccharides influence the expression of downstream antioxidant enzymes via the endogenous antioxidant stress Nrf2/ARE pathway, thereby inhibiting free radical chain reactions and reducing free radical generation (12). Furthermore, the antioxidant activity of plant polysaccharides is intricately linked to their structural characteristics (13). Research indicates that factors such as molecular weight, monosaccharide composition, glycosidic linkages, degree of branching, higher-order conformation, and chemical modifications can significantly influence the antioxidant activity of polysaccharides (14). Han et al. (5) optimized the extraction of crude polysaccharides from *Berberis dasystachya* M. using dynamic microwave-assisted extraction (DMAE) and achieved a high yield of 6.472 ± 0.384%. BDP are a class of water-soluble active polysaccharides extracted and isolated from the fruit of Huangci. The extraction processes employed can significantly influence the molecular weight distribution, structural characteristics, and biological activities of these polysaccharides (15). The crude polysaccharide extract obtained through conventional extraction methods typically contains various impurities, necessitating the application of specialized techniques for separation and purification. Commonly, the Sevag method is employed to remove proteins, while dialysis, ultrafiltration, and other techniques are utilized to eliminate small molecular impurities. Furthermore, methods such as fractional precipitation, membrane separation, and column chromatography are employed to isolate and purify polysaccharides with uniform molecular weight (16). Currently, there is a notable deficiency in the separation, purification, and structural analysis of polysaccharides derived from *Berberis dasystachya* M.

Initially, the crude polysaccharide from Nitraria spinosa was isolated and purified using DEAE-52 anion exchange column chromatography followed by dextran gel G-200 gel column chromatography. This process yielded the homogeneous polysaccharide BDP-I, whose structure was subsequently analyzed. The structural characterization of the single-component polysaccharide BDP-I was conducted employing a combination of chemical, spectroscopic, and advanced instrumental analysis techniques. The molecular weight and purity of the polysaccharide were determined through high-performance gel permeation chromatography (HPGPC). Ion chromatography was utilized to ascertain the monosaccharide composition. The glycosidic linkage patterns were elucidated using gas chromatography–mass spectrometry (GC–MS) following methylation and other derivatization procedures. Fourier-transform infrared spectroscopy (FT-IR) was employed to determine the chemical bond configurations. Furthermore, nuclear magnetic resonance (NMR) spectroscopy was used to confirm the types and proportions of glycosidic bonds, thereby elucidating the structure of the homogeneous polysaccharide BDP-I. Secondly, a stable oxidative stress model induced by H_2_O_2_ was established in RIN-m5F pancreatic cells to investigate its impact on superoxide dismutase (SOD) and catalase (CAT) activities, as well as malondialdehyde (MDA) content, in RIN-m5F pancreatic cells subjected to H_2_O_2_ damage. Subsequently, utilizing high-throughput metabolomics, the regulatory pathways involved in BDP-I intervention in oxidative damage cells were examined, alongside an investigation into the effects of BDP-I metabolites and associated metabolic pathways. In conjunction with reverse transcription polymerase chain reaction (RT-PCR) and analysis of cell apoptosis rates, the anti-apoptotic mechanisms of BDP-I on H_2_O_2_-damaged RIN-m5F pancreatic beta cells were explored.

In this study, spectroscopic, chromatographic, and mass spectrometry techniques were employed to isolate and purify crude polysaccharides from *Berberis dasystachya* M. The investigation focused on elucidating the fine structure of uniform polysaccharides BDP-I to clarify the chemical structure and active substance basis of the effective constituents in medicinal and edible berry resources from the Qinghai-Tibet Plateau. Subsequently, a chemical inducer was utilized to establish an oxidative damage model of pancreatic beta cells, facilitating an in-depth exploration of the attenuation of oxidative stress by BDP-I, at the cellular level. This research provides a crucial theoretical foundation for the effective utilization of *Berberis dasystachya* M. resources from the Qinghai-Tibet Plateau in addressing key scientific challenges. The findings are anticipated to enhance the current understanding of the mechanisms underlying the therapeutic effects of BDP-I in antioxidant activity and the treatment of Type 2 Diabetes Mellitus (T2DM), while offering valuable insights to guide the diagnosis and treatment of oxidative stress-related conditions.

## 2 Materials and methods

### 2.1 Materials

The ripe fruits of *Berberi dasystachya* M. (cultivar name: Yellow thorn) was collected in September 2023 from Datong County in Qinghai Province (latitude of 36.972, longitude of 101.576, and an elevation of 3,017 meters). Newly collected specimens were identified by Professor Shengnan Sun as NO.2023–056 for *Berberis dasystachya* M. at the Herbarium of the Department of Pharmacy, Medical College, Qinghai University, China (the species was consistent with Han et.al (2), NO. BD20140826). The herbarium samples were cataloged and deposited at the same institution. The fruits were detached from the branches, and their moisture content was reduced to approximately 13% (dry basis) through air-drying in a vacuum oven. The fruit exhibited a moisture content of 74.63%. The sugar content was relatively low, measured at 13.89%, while the crude protein content was 2.57%. Additionally, the crude fat content was determined to be 0.25%, and the crude fiber content was 17.69%. The dried fruits were characterized by a sour taste, attributable to a total acid content of 19.20%.

Monosaccharide standards (rhamnose, arabinose, mannose, fucose, xylose, glucose, etc.) were procured from Bo Rui Saccharide Biotech Co., Ltd. (Yangzhou, China), with the DEAE Sepharose Fast Flow gel and Sephadex G-200 gel acquired from the same supplier. In addition, NaH_2_PO_4_·2H_2_O, Na_2_HPO_4_·12H_2_O, potassium persulfate, NaCl, KCl, KH_2_PO_4_, and Na_2_HPO_4_ were obtained from Thermo Scientific™ Co., Ltd. (Acros Organics. Thermo Scientific, USA), while DMEM, trypsin, penicillin/streptomycin and fetal bovine serum were purchased from Gibco (Thermo Fisher Scientific Co., Ltd., USA) for cell culture. H_2_O_2_ solution, dimethyl sulfoxide (DMSO), and CCK8 were obtained from Elabscience Biotechnology Co., Ltd. (Wuhan, China, Lot no. AK80098). RIN-m5F rat pancreatic β-cells were provided by the Cell Bank of the Chinese Academy of Sciences (Shanghai, China, iCell-r029). Finally, assay kits for thiobarbituric acid reactive substances (TBARS) was obtained from Elabscience Biotechnology Co., Ltd. (Wuhan, China, Lot no. WA07XT4X05559), the Superoxide dismutase (SOD, Lot no. 20250113), and catalase (CAT, Lot no. 20241028) were obtained from Nanjing Jiancheng Technology (Nanjing, China), the Annexin IV/PI double-labeling kit were obtained from Elabscience Biotechnology Co., Ltd. (Wuhan, China, Lot no. WVO4JJF09573), while 96-well plates, 48-well plates, and 24-well plates were supplied by Corning INC. (Corning, NY, USA).

### 2.2 Separation and purification of BDP-I

The dried fruit samples were ground and sieved through a 60-mesh screen. Polysaccharide extraction from *B. dasystachya* Maxim. was extracted and preliminary membrane purification according to the method of Zhou et al. (4) to obtain uniform ones of similar molecular weights and polarities. The crude water-soluble polysaccharides were then lyophilized to a constant weight, referred to as BDP, and weighed. The yield of crude polysaccharides was 75.26 ± 3.18%, the protein content was 0.74 ± 0.06%.

The obtained membrane separated polysaccharides were purified and separated by follow steps. Briefly, the DEAE Sepharose Fast Flow filler (7.5 cm × 60 cm) was used to elution. One gram of crude polysaccharides was then dissolved in distilled water, and after being heated with constant stirring, the resulting solution was centrifuged at 12,000 rpm. The collected supernatant was washed three times with deionized water before being sequentially eluted with solutions of 0.2 M NaCl, 0.5 M NaCl, and 1.0 M NaCl at a 15 mL/min flow rate. Once the eluents (10 mL fractions per tube) were collected, the total sugar content was eventually determined by the phenol-sulfuric acid method.

Based on the characteristic profiles of the peaks, identical eluents were combined and concentrated under reduced pressure at a temperature of 45°C. The resulting solutions were then subjected to 48-h of dialysis in a 3,500 Da dialysis tubing, after which they were freeze-dried and sealed for storage. These samples were labeled as BDP-W, BDP-I, BDP-II, and BDP-III. One hundred milligrams of polar-separated polysaccharides (BDP-I) was dissolved in 3 mL of distilled water, and after centrifugation at 12,000 rpm for 10 min, the resulting supernatant was purified using a Sephadex G-200 gel permeation column (2.6 cm × 60 cm). The purified sample was then collected by high-performance liquid chromatography, which was equipped with a refractive index detector (RID-10A, Shimadzu, Tokyo, Japan) that enabled the generation of a symmetrical peak and an elution curve (17). The individual components were finally concentrated under reduced pressure at 45°C, and after being freeze-dried, the protein content was determined to be 0.36 ± 0.02% by the Coomassie Brilliant Blue method. Then these purified gel column-separated polysaccharides were subjected to physical and chemical analyses.

### 2.3 Characterization of BDP-I

#### 2.3.1 Molecular weight determination

The molecular weight of BDP-I was assessed using HPGPC. The analysis was conducted on an LC-10A HPLC system (Shimadzu, Tokyo, Japan) equipped with a BRT105-104-102 series column of 8 mm × 300 mm. In addition, a standard curve of dextran was drawn, with the retention time (x, min) of each standard on the horizontal axis and the logarithmic value of its molecular weight (y, Log MW) on the vertical axis. The molecular weight of the polysaccharide was then determined from the best fit of the regression equation.

#### 2.3.2 Infrared spectral determination

After precisely weighing 2 mg of the dried sample and 200 mg of potassium bromide, the two substances were combined and applied to the surface of the diamond prism in the attenuated total reflection configuration, followed by compression. Tablets composed solely of potassium bromide powder served as the blank control. Subsequently, the samples were analyzed using a Fourier transform infrared spectrometer (FTIR 650, Tianjin Gangdong Co., Hebei, China). Data analysis was performed using SpectraGryph version 1.2 software (Oberstdorf, Germany).

#### 2.3.3 Monosaccharide composition

Standard solutions of 16 monosaccharides were prepared using a concentration gradient of 0.1 to 50 mg/L. A sample of 5 mg was carefully deposited into an ampoule and combined with 10 mL of a 2 M trifluoroacetic acid solution. The mixture was then subjected to hydrolysis at a temperature of 120°C for 3 h and then transferred to a tube for drying under a stream of nitrogen. The resulting solution (100 μL) was combined with 900 μL of deionized water. After centrifugation at 12,000 rpm for 5 min, the supernatant was collected for monosaccharide analysis on a Dionex ICS-5000 system (Thermo Scientific Co., Waltham, MA, USA) equipped with a CarboPacTMPA-20 analytical column (3 mm × 150 mm).

#### 2.3.4 Methylation and GC–MS determination

Following the methylation, hydrolysis, and acetylation procedures, the GC–MS technique was employed to determine and compare the obtained results with the standard mass spectrum library. The polysaccharide sample was first weighed (50 mg) and dissolved in anhydrous DMSO before initiating the methylation reaction by adding the methylating reagent solution. For hydrolysis, TFA was added to the sample, followed by the addition of sodium borohydride for reduction. After cooling, acetic anhydride was added for acetylation, with the resulting mixture added to toluene. Vacuum concentration and drying were then performed, and this process was repeated 4–5 times to eliminate excess acetic anhydride. Finally, the acetylated product was dissolved in dichloromethane. All analyses were performed with the GC–MS instrument (GCMS-QP-2010 model, Shimadzu, Tokyo, Japan).

#### 2.3.5 Nuclear magnetic resonance spectroscopy

The polysaccharide sample was measured (50 mg) and dissolved in 0.5 mL of deuterated water (D_2_O) with a purity of 99.9%. After thorough dissolution, freeze dry and repeat the exchange three times. Dissolve the processed sample in D_2_O again, centrifuge and transfer the solution to a 5 mm NMR tube, then the resulting solution was then freeze-dried for spectral analysis with a 600 MHz spectrometer (Bruker Corp. Fallanden, Switzerland) equipped with a 5-mm probe. Both 1D (1H, 13C) and 2D (COSY, TOCSY, HSQC, HMBC) spectra were acquired, with the results subsequently analyzed using Mestre Nova 14 software.

### 2.4 RIN-m5F cell culture

The protocols for cell culture, passaging, cryopreservation, and recovery of RIN-m5F pancreatic islet cells were adapted from the methodology outlined by Luo et al. (18).

### 2.5 Effects of H_2_O_2_ on RIN-m5F cells

#### 2.5.1 Cell viability assay

A cell suspension (100 μL), at a density of 1 × 105 cells/mL, was seeded into each well of 96-well plates before a 24-h incubation to allow cell attachment. Different concentrations of H_2_O_2_ (0 ~ 500 μM) were added to the culture plates before incubation for 3, 6, 12, and 24 h, respectively, to induce oxidative damage. The culture medium was discarded, and 100 μL of CCK-8 solution (CCK-8 reagent: serum-free culture = 1:10) was added to each well. Six replicates were prepared for each experimental group to ensure the accuracy of the results. After adding the CCK-8 solution, the plates were incubated at a constant temperature for 0.5 h to allow the reagent to react with the cells. The cell survival rate was subsequently calculated as Equation 1:


(1) $$\text{Cell survival rate}\;(\%)=\frac{A_{1}-A_{2}}{A_{3}-A_{2}}\times 100\%$$

Where *A*_1_ represents the absorbance value of the experimental group, *A*_2_ represents the absorbance value of the blank group, and *A*_3_ represents the absorbance value of the normal control group (NC).

#### 2.5.2 Intracellular ROS scavenging activity

The 2′,7′-dichlorodihydrofluorescein diacetate (DCFH-DA) fluorescence probe method was employed to assess the levels of reactive oxygen species (ROS). In this experiment, the fluorescence intensity was quantified utilizing an enzyme marker analyzer with an excitation wavelength of 488 nm and an emission wavelength of 525 nm.

### 2.6 Protective effects on high-glucose and H_2_O_2_-induced RIN-m5F cell injury

#### 2.6.1 Selection of BDP-I concentration

A cell suspension (100 μL), at a density of 1 × 10^5^ cells/mL, was added to each well of a 96-well plate and allowed to attach for 24 h. Different concentrations of BDP-I (0.0625, 0.125, 0.25, 0.50, 1, and 2 mg/mL) were then added, and after incubation for 24, 48, and 72 h, respectively, the CCK-8 method was used to detect cell survival rate to determine the optimal concentration range of BDP-I.

Based on the results of cell survival, the following groups could be identified: the NC, where RPMI-1640 medium containing 10% fetal bovine serum was used, the positive control group (PC) treated with 300 μmol/L of *α*-LA, the model group (MC) treated with 250 μmol/L of H_2_O_2_ as well as the BDP-I-treated groups which could be further subdivided into the BDP-I low-dose group (0.0625 mg/mL), BDP-I medium-dose group (0.125 mg/mL) and BDP-I high-dose group (0.25 mg/mL).

#### 2.6.2 Effects of BDP-I on the survival rate and ROS level of oxidative damaged cells

Following the successful establishment of the oxidative stress model, different concentrations of BDP-I (0.0625, 0.125, 0.25 mg/mL) were used for treatment to assess its effects. Each group had six replicates, and both the blank group and the MC were cultured under identical conditions as the BDP-I-treated groups. The cells were then incubated for 24 h after treatment, with the survival rate of RIN-m5F cells eventually assessed as described in section 2.5.1. In addition, the ROS level was determined, as mentioned in section 2.5.2.

#### 2.6.3 Effects of BDP-I on the antioxidant activity of oxidative damaged cells

In this experiment, the cells were grouped and treated as described in section 2.6.1. Following treatment, the cells were collected, rinsed with PBS, centrifuged, homogenized using an extraction solution, and eventually pulverized through electric grinding for 10 s per iteration, followed by a 10-s pause in between for eight cycles. Antioxidant activity was finally assessed according to the instructions provided in the SOD, CAT, and TBARS test kits, the TBARS values were then expressed as equivalent nanomoles of malondialdehyde (MDA).

### 2.7 Cell metabolomics

#### 2.7.1 Cell sample collection and preparation

The cells were incubated in 3-cm dishes as described in section 2.6.1. Following three washes with 0.9% saline solution, they were quenched in liquid nitrogen for 3 min, after which an ice-cold 80% methanol: water (pre-cooled) solution was added. The cells were finally scraped off with a cell scraper.

#### 2.7.2 Sample extraction

An appropriate amount of the sample was added to a methanol/acetonitrile/water solution (2:2:1, v/v). After ultrasounds at low temperatures for 30 min, the sample was then centrifuged and vacuum drying the resulting supernatant. The collected was re-dissolution and eventually injected for mass spectrometry analysis.

#### 2.7.3 UHPLC-Q-TOF-MS (chromatography-mass spectrometry) analysis

A liquid chromatography system with ultra-high performance (Agilent, USA) was used to separate the samples. An automatic injector was used to inject the sample at 4°C, along with QC samples added to the sample queue to monitor and assess the system’s stability and data reliability.

An AB Triple TOF 6600 mass spectrometer (Agilent, USA) was used to collect the first and second spectra of the sample, with the positive and negative ion modes of electrospray ionization (ESI) subsequently used for detection. Data-dependent acquisition mode (IDA) and peak intensity value screening mode were used for the second mass spectrometry.

### 2.8 Real-time quantitative polymerase chain reaction (RT-qPCR)

In order to study the molecular mechanisms through which BDP-I could prevent oxidative damage of pancreatic islet cells, qRT-PCR was performed to determine the genes involved. For this purpose, RNA was first extracted from samples using Trizol reagent (Invitrogen, Carlsbad, CA, USA) and quantified with a UV spectrophotometer (Thermofisher, USA). Total RNA was then reverse-transcribed into cDNA before amplification using a reverse transcription kit (Takara, Dalian, China) and specific primer pairs (Supplementary Table S1). Quantification was achieved by incorporating SYBR Green using Rotor-Gene Q (Qiagen, Duesseldorf, Germany), with the levels of gene expression eventually assessed by comparative threshold cycle analysis against the GAPDH gene as an internal control.

### 2.9 Cell apoptosis rate

Remove the culture medium from each cell group, and subject the cells to digestion using 0.25% trypsin. Examine the cells under an inverted phase contrast microscope. Upon observing the retraction of pseudopodia and the rounding of cells, promptly remove the trypsin solution and introduce a culture medium supplemented with 10% fetal bovine serum (FBS) to halt the digestion process. Gently agitate the cells to ensure even dispersion, and collect the resulting cell suspension. Proceed with centrifugation to harvest the cells. Subsequently, apply the Annexin IV/PI double-labeling kit to the cells for reaction. The rate of apoptosis is then quantified using flow cytometry.

### 2.10 Statistical analysis

One-way analysis of variance (ANOVA) was performed to compare multiple groups to determine whether the NC differed from the BDP-I-treated group. The quality of the normalized data was also assessed before multivariate statistical analysis, which included principal component analysis (PCA) and orthogonal partial least squares discriminant analysis (OPLS-DA). In the latter case, the variable importance values (VIP > 1, SPSS significance analysis *p* < 0.05) were used to screen for differential metabolites between groups. The m/z values of the compounds were also entered into the HMDB database for metabolite screening, with the HMDB ID of any significant differential metabolites subsequently entered into the MetaboAnalyst database for further validation as well as for bioinformatics analysis, which included clustering analysis, correlation analysis, pathway analysis, amongst others.

## 3 Results and discussion

### 3.1 The isolation and purification of BDP-I

**Figure 1:**
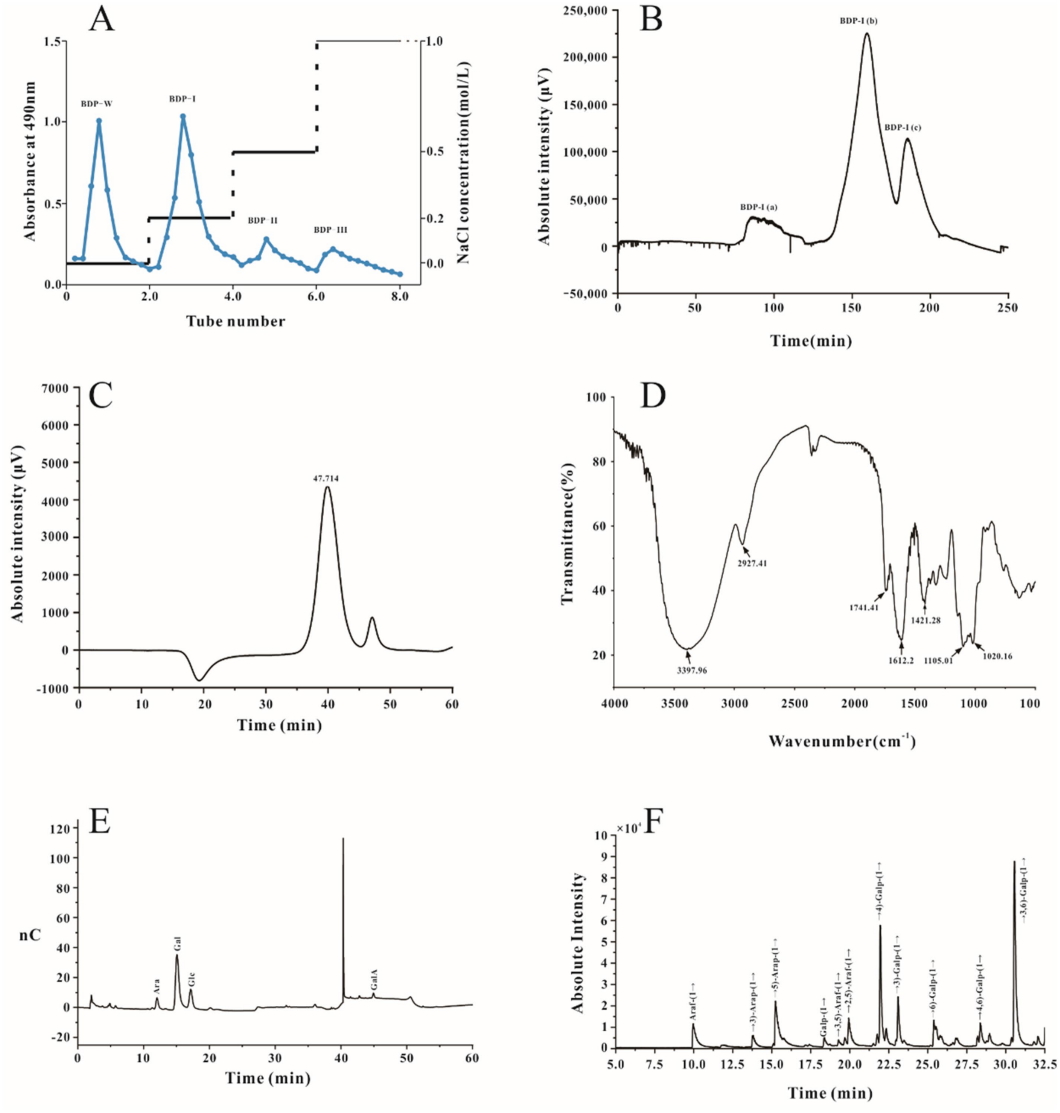
The elution profiles and characterization of BDP-I. **(A)** The elution curve of DEAE anion column chromatography DEAE; **(B)** Sephadex G-200 column chromatography; and characterization of polysaccharide fractions **(C–F)**, **(C)** HPGPC chromatogram of BDP-I; **(D)** FT-IR spectrum of BDP-I; **(E)** GC chromatography of BDP-I; **(F)** The GC–MS chromatogram of PMAAs of BDP-I.

Crude polysaccharides were extracted from *Berberis dasystachya* M. (BDP) using dynamic microwave-assisted extraction, with the yield being 6.76 ± 0.062% (w/w). The extracted polysaccharides were then purified through ethanol precipitation, dialysis, and lyophilization before DEAE Sepharose Fast Flow chromatography, which applied gradients of 0, 0.2, 0.1, and 1.0 mol/L NaCl solutions for further purification. The eluted fractions were then collected and designated as BDP-W, BDP-I, BDP-II, and BDP-III (Figure 1A). The distilled water (BDP-W, 13.6%) and 0.2 M NaCl fractions (BDP-I, 20.45%) were selectively collected by the fraction collector, while the one eluted with 0.5 M sodium chloride was not collected due to its lower collection rate (BDP-II, 3.89%; BDP-III, 0.72%). Previous studies have extensively investigated the properties of BDP-W, so the current study focused specifically on BDP-I. After additional dialysis and lyophilization, BDP-I was subjected to Sephadex G-200 column chromatography (Figure 1B), resulting in the separation of BDP-I into three homogeneous polysaccharides, namely BDP-I (a) (5.82%), BDP-I (b) (74.07%), and BDP-I (c) (19.75%). In this case, BDP-I (b) exhibited a larger peak area than BDP-I (a) and BDP-I (c), suggesting that it was the major component in BDP-I. Subsequent analysis was conducted on concentrated, purified, and lyophilized BDP-I (b).

Polysaccharides, as intricate and diverse macromolecular entities, exhibit their complex structures during the investigation of their biological activities, which serves as a fundamental basis for examining structure–activity relationships. Currently, the methodologies for extracting plant polysaccharides have reached a level of maturity and primarily include traditional water extraction, ultrasound-assisted extraction, enzymatic extraction, microwave-assisted extraction, and supercritical fluid extraction, among others (19). The extraction process for most plant polysaccharides necessitates sample pretreatment steps, such as impurity removal, defatting, and decolorization (20), followed by the selection of appropriate extraction techniques to obtain polysaccharide solutions, which may still contain impurities. To achieve polysaccharides with a uniform composition, further separation and purification are essential. The choice of separation and purification methods is intricately linked to subsequent structural analysis and research into the biological activities of polysaccharides.

Column chromatography is a widely utilized technique for separation and purification in laboratory settings. It is particularly effective in enhancing the purity of plant polysaccharides. Various methods of column chromatography include gel filtration, ion exchange chromatography, affinity chromatography, and reverse phase chromatography (21). Currently, commonly employed anion exchange columns consist of DEAE cellulose, DEAE glucan gel, and DEAE agarose gel, with DEAE cellulose columns being predominantly used for polysaccharide purification. Polysaccharide components isolated via DEAE anion exchange columns are subsequently subjected to further separation and elution using gel columns, ultimately yielding homogeneous polysaccharide components. Frequently used gel columns include Sephadex, Sepharose, Sephacryl, and Superdex, with Sephadex being particularly prevalent in polysaccharide separation and purification processes. According to literature, crude polysaccharides extracted from pumpkin were purified using DEAE Sepharose Fast Flow and Sephadex G-100 columns to obtain homogeneous polysaccharides (22). A novel polysaccharide from Macadamia peel was purified with DEAE-52 and Sephadex G-50 columns, showed significant antioxidant activity with respect to DPPH, hydroxyl group and reducing power (23). Consequently, the purification of polysaccharides via column chromatography to achieve homogeneous component polysaccharides is advantageous for the advanced analysis of polysaccharides. This approach facilitates a structural foundation for investigating the activity and intricate structure–activity relationships of plant polysaccharides.

### 3.2 The structural characteristics of BDP-I

#### 3.2.1 Homogeneity and molecular weight analysis

To determine the purity and molecular weight of the purified homogeneous yellow thorn polysaccharide BDP-I, HPGPC was performed, with glucose standards of known molecular weights ranging from 5,000 Da to 3,693,000 Da also included for the experiment. As illustrated in Figure 1C, the elution curve exhibited a single absorption peak characterized by favorable peak symmetry, reflecting the BDP-I sample’s high purity. Based on the glucose standard curve, BDP-I’s peak molecular weight was subsequently established as 3.947 kDa, with its weight-average molecular weight and number-average molecular weight being 4.771 kDa and 3.264 kDa, respectively. Compared to the study by Yang et al. (24), a homogeneous acidic polysaccharide (RPP-2a) was isolated from Raspberry Pulp, with a low weight-average molecular weight (Mw) of 55.582 KDa, BDP-I could be classified as a low-molecular-weight polysaccharide.

#### 3.2.2 FT-IR spectroscopy of BDP-I analysis

As illustrated in Figure 1D, the infrared spectrum of BDP-I exhibited an absorption peak within the range of 3,600–3,200 cm^−1^, indicative of the -OH stretching vibration, a characteristic feature of carbohydrates. Similarly, an absorption peak was observed at 3300–3500 cm^−1^, corresponding to the O-H stretching vibration, another distinctive absorption feature of carbohydrates. A weaker absorption peak was detected near 2930.1 cm^−1^, attributed to the C-H stretching vibration on the sugar chain, which is recognized as one of the characteristic peaks of polysaccharides. Furthermore, the absorption peaks at 2927.41 cm^−1^ and 2927.90 cm^−1^ indicated the presence of asymmetric bending vibrations of C-H bonds, also characteristic of polysaccharides. The stretching vibration of C-H was also visible as a distinct absorption peak at 2927 cm^−1^, while a peak at 1740 cm^−1^ indicated the stretching vibration of C=O, which reflected the presence of acetyl groups within the BDP-I structure. Maldonado demonstrated that high methoxylation (degree of methoxylation, DM > 70%) pectin standards exhibit signals at 1630 and 1736 cm^−1^, whereas low esterification (DM < 30%) pectin standards only display a band at 1593 cm^−1^. The BDP-I pectin exclusively presented the carboxylate ion signal near 1730 cm^−1^, suggesting a high degree of methoxylation (25). The absorption peak at 1612 cm^−1^ may serve as a characteristic peak of crystalline water. A weak absorption peaks were evident at 1540 cm^−1^ in the IR spectrogram, indicating that the polysaccharides contained a few proteins component. In addition, a peak at 1421 cm^−1^ could be attributed to the stretching vibrations of C-O. Furthermore, absorption peaks in the range of 1,200–1,000 cm^−1^ indicated the presence of pyranose structures. In this region, the band −1 at 1011 cm^−1^ corresponds to the vibrational characteristics of the *α* − 1-4-C-O-C bond of galacturonic acid, and its intensity is roportional to the degree of polymerization. Finally, absorption peaks at 1105 cm^−1^ could be linked to O-H bending vibrations, while absorption at 1020 cm^−1^ could be attributed to pyranose ring stretching vibrations (26).

#### 3.2.3 Monosaccharide composition of BDP-I

As illustrated in Figure 1E, compared with the HPLC chromatogram of monosaccharide standards, the monosaccharide composition of the purified polysaccharide included arabinose (Ara), galactose (Gal), glucose (Glc), and galacturonic acid (GalA). The molar ratio of each monosaccharide was determined by comparing its retention time and response intensity with those of various standard samples, with the results indicating that BDP-I was made up of Ara: Gal: Glu: GalA = 108:99:89:704.

In the previous work of the research, the monosaccharide results showed that the main monosaccharide of BDP was mannose (4). Mannose, a significant monosaccharide, frequently assumes a pivotal role in the architecture of polysaccharides. Notably, mannose is integral to the composition of plant cell wall polysaccharides, particularly in the biosynthesis of specific legume starches and fungal polysaccharides (27). The study found no mannose in BDP-1, likely due to the selectivity of polysaccharide purification and the sensitivity of analytical methods. We suspect there are two main reasons for this: Firstly, mannose may have beewith other components, or it may not have been sufficiently enriched during selective elution. Secondly, widely employed analytical techniques, including high-performance liquid chromatography (HPLC) and gas chromatography (GC), often exhibit inadequate sensitivity for the detection of mannose. This limitation is particularly pronounced in complex polysaccharide mixtures, where the presence of other components can interfere with the mannose signal, leading to its potential non-detection.

#### 3.2.4 GC–MS analysis of methylation

Due to aldonic acid’s presence in BDP-I, aldonic acid reduction was performed before methylation. The monosaccharide composition of the resulting sample was checked to confirm the successful reduction of aldonic acid (28).

Following methylation, hydrolysis, and acetylation of BDP-I, the resulting products included partially methylated alditol acetate derivatives (PMAAs). The GC–MS total ion chromatogram of these PMAAs is depicted in Figure 1F. Identification of the sugar residues present in BDP-I was achieved by comparing the mass spectra of each PMAA chromatographic peak with the established PMAA spectra of sugar residues available in the online database of the Complex Carbohydrate Research Center. Quantitative analysis was subsequently conducted by assessing the peak areas, enabling the types and molar percentages of methylated sugar residues to be determined (Table 1).

**Table 1:** Methylation analysis results of BDP-I.

Retention time (min)	Methylated sugar	Molar ratios/%	Linkages patterns
9.977	2,3,5-Me3-Araf	7.40	1-linked Araf
13.815	2,4-Me2-Arap	2.98	1,3-linked Arap
15.243	2,3-Me2-Araf	10.37	1,5-linked Araf,
18.371	2,3,4,6-Me4-Galp	1.60	1-linked Galp
19.279	2-Me1-Araf	1.24	1,3,5-linked Araf
19.932	3-Me1-Araf	5.64	1, 2,5-linked Araf
21.957	2,3,6-Me3-Galp	18.03	1,4-linked GalAp
23.099	2,4,6-Me3-Galp	6.77	1, 3-linked Galp
25.395	2,3,4-Me3-Galp	7.52	1, 6-linked Galp
28.362	2,3-Me2-Glcp	3.45	1, 4,6-linked Glcp-
30.562	2,4-Me2-Galp	35.00	1,3,6-linked Galp

The glycosidic linkage pattern of BDP-I was found to be complex, with 11 different glycosidic linkage forms. Specifically, arabino-furanose (Araf) exists in five forms: terminal sugar (1-Araf), the 1,5-Araf, the 1,3,5-Araf, the 1,2,5-Araf, and the 1,3-Arap. Similarly, Gal exists in five forms, namely the terminal sugar (1-Galp), the 1,4-GalAp, the 1,3-Galp, the 1,6-Galp, and the 1,3,6-Galp, while for Glc, only the 1,4,6-Glcp is present. Overall, GalA and Gal had the highest proportion, with 1,4-GalAp and 1,3,6-Galp accounting for 18.03 and 35.00% of the samples, respectively. Additionally, the amount of 1,5-linked Araf, 1,4-GalAp, and 1,3,6-Galp residues was 10.37, 18.03, and 35.00%, respectively, thereby suggesting that the main chain of BDP-I may be connected by 1,6-O or 1,4-O glycosidic linkages.

Galacturonic acid (GalA) is a crucial polyhydric acid predominantly found in plant pectin. Recent studies have demonstrated that GalA not only contributes significantly to the structural integrity of plant cell walls but also exhibits notable antioxidant properties and provides protection against cellular oxidative stress. Research indicates that polysaccharides abundant in GalA, such as acidic polysaccharides derived from various plant components, possess substantial antioxidant activity. These polysaccharides enhance cellular antioxidant capacity by augmenting total antioxidant capacity and the activities of superoxide dismutase and catalase, while concurrently reducing intracellular levels of reactive oxygen species and malondialdehyde (29). Furthermore, studies have revealed that GalA and its derivatives can stimulate the proliferation of human skin fibroblasts and offer a degree of protection against oxidative damage (30). Arabinose (Ara) is a significant plant-derived monosaccharide. Polysaccharides extracted from prickly pear leaves, which contain arabinose, demonstrate notable antioxidant and alpha-glucosidase inhibitory activities *in vitro*, suggesting their potential as natural hypoglycemic or antioxidant agents (31). The interaction of arabinose with arabinose A and L-ascorbic acid (vitamin C) results in a pronounced synergistic antioxidant effect. This combination not only enhances the survival rate and antioxidant enzyme activity of HEK293 cells exposed to hydrogen peroxide but also mitigates the release of lactate dehydrogenase (LDH) and the accumulation of lipid peroxidation products (32).

The structure and properties of glycosidic bonds have a significant impact on their biological activity. Two polysaccharides (DGS1 and DGS2) isolated from Dendrobium officinale contain glycosidic bonds of (1 → 5)—Alaf, (1 → 4)—Glcp, and (1 → 6)—Glcp. These polysaccharides activate the expression of TNF-*α*, IL-6, and iNOs genes, particularly DGS2, which significantly increases the levels of TNF-α, IL-6, and NO, demonstrating stronger antioxidant activity (33). Polysaccharides rich in 1,3,6-Galp residues exhibit various biological activities, such as a water-soluble neutral polysaccharide (CAPW-1) that was considered of 1,3-Galp and 1,3,6-Galp, could stimulate the proliferation of RAW264.7 cells and promote the secret of nitrogen oxide (NO), interleukin-6 (IL-6), and tumor necrosis factor-*α* (TNF-α) with no cytotoxicity (34).

#### 3.2.5 NMR spectra analysis

**Figure 2:**
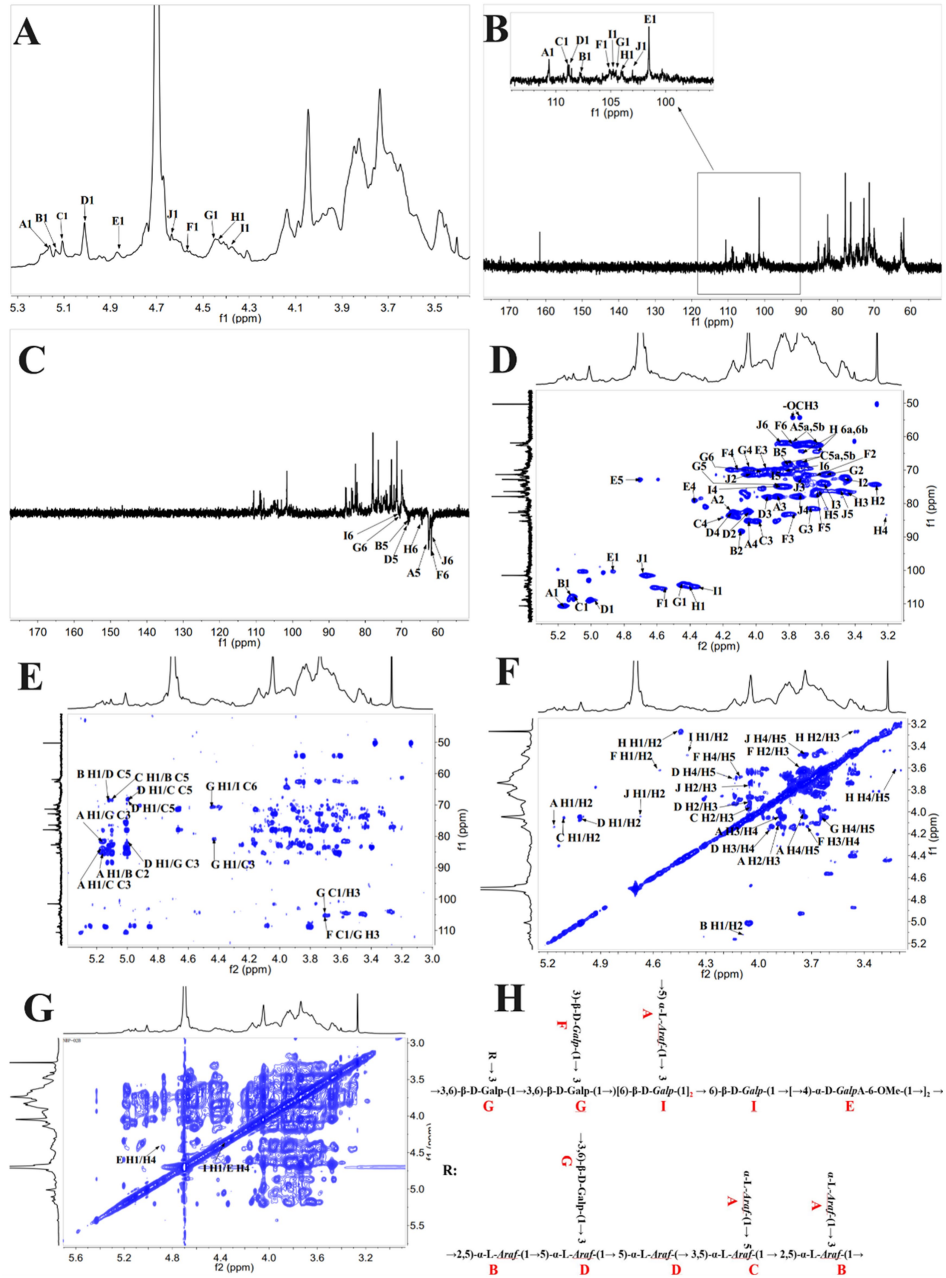
NMR spectra and the suggested structure of BDP-I. **(A)** ^1^H NMR; **(B)** ^13^C HMR; **(C)** DEPT 135; **(D)** ^1^H-^1^H COSY; **(E)** HSQC; **(F)** NOESY; **(G)** HMBC and **(H)** suggested structure of BDP-I.

To obtain the configuration and linkage information of the sugar residues, the 1D/2D NMR spectra of BDP-I were analyzed (Figure 2), and these included 1H NMR, 13C NMR, COSY, TOCSY, NOESY, HSQC, and HMBC spectra. Interestingly, some of the signals were consistent with those reported in studies, hence establishing them as β-D-Galp and α-L-Araf (24, 35).

##### 3.2.5.1 1D/2D NMR spectrum analysis

Further analysis of the structure of BDP-I was achieved using 1D NMR (C/H spectra) and 2D NMR (HSQC/HMBC), with the 1H-NMR and 13C-NMR spectra shown in Figures 2A,B, respectively. Figure 2A illustrates that the proton signals of BDP-I predominantly resided within the range of 3.0 to 6.0 ppm. On the other hand, the proton peaks originating from the terminal groups, specifically at δ5.17, 5.14, 5.11, 5.01, 4.94, 4.71, 4.65, 4.55, 4.47, 4.4 and 4.37, were dispersed within the 4.3–6.0 ppm region. The chemical shifts of these terminal protons fall below 5.0 ppm and exceed 5.0 ppm, and hence, these results suggested the coexistence of *α* and β configurations of glycosidic linkages in BDP-I as supported by the methylation results and prior literature (36).

By analyzing the 13C-NMR spectrum, the chemical shifts of the corresponding terminal carbons can be determined. It was noted that the carbon signals of the terminal groups in BDP-I were predominantly concentrated within the range of 100 to 110 ppm (Figure 2B). Specifically, there were 10 distinct carbon signal peaks denoted as A, B, C, D, E, F, J, H, and I, and they were located at δ110.62, 108.89, 108.88, 107.8, 105.39, 104.9, 104.69, 104.48, 101.49 and 100.38 ppm, respectively. However, the methylation results showed that BDP-I had 11 different sugar residues, and these differences in signals could be attributed to the masking of signals from sugar residues with very low content (37). By comparison, it was found that the chemical shifts of C-1 in A, B, C, D, and I were around 100 ppm, while those in E, F, J, and H were close to 107 ppm, indicating that they belonged to sugar residues from three different monosaccharide sources. Combined with the results of monosaccharide composition, Glc, Gal, and Ara were identified as the main ones. Furthermore, analysis of the DEPT-135 spectrum (Figure 2C) revealed reverse peaks at δ62.64, 68.27, 67.79, 68.25, 70.76, 70.5 and 61.6, indicating signal peaks from C6 or C5.

The proton signals corresponding to the quaternary carbons in the spectrum were obtained through a combination of 1H-1HSQC spectrum (Figure 2D) and 1H-NMR analysis. The chemical shifts, in this case, were 5.17, 5.01, 5.11, 5.14, 4.47, 4.37, 4.55, 4.4, 4.65, and 4.94 ppm, and more specifically δ110.62/5.17, 108.88/5.01, 108.89/5.11, 107.8/5.14, 104.69/4.47, 104.9/4.37, 105.39/4.55, 104.48/4.4, 101.49/4.65, and 100.38/4.94. Additionally, the signal at δC 54.5 was attributed to the -OCH3 of *α*-GalpA, and the signal at δH3.74/δC54.5 was assigned to the methyl group on O-2/3 of α-GalpA. According to the methylation analysis results and literature findings, these signals were assigned to glycosidic linkages: α-L-Araf-(1→, →2,5)-α-L-Araf-(1→, →3,5)-α-L-Araf-(1→, →3,6)-β-D-Galp-(1→, →6)-β-D-Galp-(1→, →3)-β-D-Galp-(1→, β-D-Galp-(1→, →4)-β-D-Galp-(1→, →4)-α-D-GalAp-(1→ (24), corresponding to A, B, C, D, E, F, G, H, I and J in the HSQC spectrum. Further resolution was achieved by combining the HSQC, ^1^H-^1^H COSY, HMBC, and NOESY spectra of BDP-I (Figures 2D–G) with 1H-NMR and HSQC spectra, as well as the carbon-hydrogen assignments for each cross-peak are detailed in Table 2.

**Table 2:** Assignment of 1H-NMR and 13C-NMR chemical shifts of BDP-I monosaccharide residues.

Monosaccharide residues glycosyl residues	Chemical shift (δ, ppm)							
	Type	H1/C1	H2/C2	H3/C3	H4/C4	H5/C5	C6/H6a,b	-OCH_3_
A α-L-Araf-(1→	H	5.17	4.13	3.87	4.06	3.76	3.64	
	C	110.62	82.62	77.97	85.22	62.64		
B 2,5)-α-L-Araf-(1→	H	5.14	4.08	4.18	3.93	3.8		
	C	107.8	88.5	78.3	83.75	68.25		
C → 3,5)-α-L-Araf-(1→	H	5.11	4.2	3.99	4.23	3.8	3.75	
	C	108.89	80.6	83.64	83.29	67.79		
D → 5)-α-L-Araf-(1→	H	5.01	4.07	3.94	4.15	3.82;3.75	3.7	
	C	108.88	82.18	78.12	83.68	68.27		
E → 4)-α-D-GalpA-6-OMе-(1→	H	4.87	3.67	3.93	4.37	4.71		3.73
	C	101.51	69.4	70.05	79.15	72.65	172.33	54.13
F → 3)-β-D-Galp-(1→	H	4.55	3.63	3.77	4.12	3.67	3.75	
	C	105.39	72.1	83.22	69.74	76.9	61.6	
G → 3,6)-β-D-Galp-(1→	H	4.47	3.57	3.68	4.05	3.87	3.96	3.86
	C	104.69	71.31	81.5	69.82	74.81	70.76	
H β-D-Galp-(1→	H	4.4	3.28	3.46	3.21	3.6 0	3.72	3.64
	C	104.48	74.66	76.62	83.4	77.36	64	
I → 6)-β-D-Galp-(1→	H	4.37	3.44	3.58	3.86	3.88	3.95	3.83
	C	104.9	72.16	73.93	74.96	69.87	70.5	
J → 4)-β-D-Galp-(1→	H	4.65	4.02	3.7	3.71	3.45	3.8	3.71
	C	101.49	71.39	72.81	77.8	76.2	61.69	

##### 3.2.5.2 Residue analysis

From the three spectra of cross-peak A (Figures 2E–G), the quaternary carbon signal was observed at δ110.62, and the corresponding quaternary proton signal in the HSQC spectrum was at δ5.01. In addition, through 1H-1H COSY analysis, the signals for H1/H2 were at 5.17/4.13 ppm. According to the COSY spectrum (Figure 2E), the cross-peak of signals at δ5.17 ppm and δ4.13 ppm indicated that the chemical shift of H-1 in residue A is δ5.17 ppm, corresponding to C1 at 110.62. Sequentially, the signals for H2/H3 are at 4.13/3.87 ppm; H3/H4 signals are at 3.87/4.0 ppm; H4/H5 signals are at 4.0/3.76 ppm, and H5/H6ab signals are at 3.76/3.64 ppm. By analyzing the adjacent proton correlations in the COSY spectrum, it was inferred that H2, H3, H4, H5, and H6ab corresponded to δ5.17, 4.13, 3.87, 4.06, 3.76, 3.64, respectively, while the corresponding C2-C5 was 82.17, 78.11, 83.67, 68.26. The carbon and hydrogen chemical shift assignments for residue A in BDP-I are determined as →5) *α*-L-Araf-(1→. From the three spectra of cross-peak B, the quaternary carbon signal is observed at δ107.8, and the corresponding quaternary proton signal in the HSQC spectrum is at δ5.14. Through 1H-1H COSY analysis, the chemical shift of H-1 in residue B is δ5.14 ppm, corresponding to C1 at 107.8. Similarly, the chemical shifts for H-2, H-3, H-4, and H-5 are δ4.08 ppm, 4.18 ppm, 3.93 ppm, and 3.8 ppm, respectively, and the corresponding C2-C5 are 88.5, 78.3, 83.75, 68.25 (38). The carbon and hydrogen chemical shift assignments for residue B in BDP-I are determined as →2,5)-*α*-L-Araf-(1 → .

From the three spectra of cross-peak C, the quaternary carbon signal was observed at δ108.89, and the corresponding quaternary proton signal in the HSQC spectrum was at δ5.11. Through 1H-1H COSY analysis, the chemical shift of H-1 in residue C was δ5.11 ppm, corresponding to C1 at 108.89. Similarly, the chemical shifts for H-2, H-3, H-4, H-5, H-6 were δ4.20 ppm, 3.99 ppm, 4.23 ppm, 3.8 ppm, and 3.75 ppm, while the corresponding C2-C5 were 80.6, 83.64, 83.29, 67.79. The carbon and hydrogen chemical shift assignments for residue C in BDP-I were determined as →3,5)-*α*-L-Araf-(1→ (39, 40). From the three spectra of cross-peak D, the quaternary carbon signal was observed at δ108.88, and the corresponding quaternary proton signal in the HSQC spectrum was at δ5.01. Through 1H-1H COSY analysis, the chemical shift of H-1 in residue D is δ5.01 ppm, corresponding to C1 at 108.88. Similarly, the chemical shifts for H-2, H-3, H-4, H-5a; 5b, H-6 were δ4.07 ppm, 3.94 ppm, 4.15 ppm, 3.82; 3.75 ppm, 3.7 ppm. The corresponding C2-C5 were 82.18, 78.12, 83.68, 68.27. The carbon and hydrogen chemical shift assignments for residue D in BDP-I are determined as →5)-*α*-L-Araf-(1 → .

Using the analysis mentioned above and drawing upon relevant literature findings (18, 26, 41–43), the assignment of carbon and hydrogen chemical shifts were sequentially determined for residue E as BDP-I as →4)-α-D-GalpA-6-OMe-(1→; for residue F as →3)-β-D-Galp-(1→; for residue Gas →3,6)-β-D-Galp-(1→; for residue H as β-D-Galp-(1→; for residue I as →6)-β-D-Galp-(1→; and for residue J as →4)-β-D-Galp-(1 → .

Therefore, comprehensive analysis revealed the carbon and hydrogen chemical shift assignments for monosaccharide residues in BDP-I (Table 2). Taken together, the results indicated that BDP-I contained 10 types of monosaccharide residues, including →5)-*α*-L-Araf-(1→, →5)-α-L-Araf-(1→, →3,5)-α-L-Araf-(1→, →2,5)-α-L-Araf-(1→, →3,6)-β-D-Galp-(1→, →6)-β-D-Galp-(1→, →3)-β-D-Galp-(1→, β-D-Galp-(1→, →4)-β-D-Galp-(1→, →4)-*α*-D-GalAp-(1 → .

##### 3.2.5.3 Main chain analysis

In the HMBC spectrum, the quaternary proton (4.47 ppm) of →3,6)-β-D-Galp-(1 → (G) showed correlation signals with its own C3 (81.5 ppm); simultaneously, the quaternary carbon (104.69 ppm) of →3,6)-β-D-Galp-(1 → (G) showed correlated signals with its own H3 (3.68 ppm). This indicates the presence of a linkage pattern →3,6)-β-D-Galp-(1 → 3,6)-β-D-Galp-(1 → (G → G). In the HMBC spectrum, the quaternary proton (4.47 ppm) of →3,6)-β-D-Galp-(1 → (G) also showed correlated signals with the C6 (70.5 ppm) of →6)-β-D-Galp-(1 → (I). The results therefore suggested the presence of a linkage pattern →3,6)-β-D-Galp-(1 → 6)-β-D-Galp-(1 → (G → I) (44).

In the NOESY spectrum, the quaternary proton (4.37 ppm) of →6)-β-D-Galp-(1 → (I) exhibited correlated signals with the CH4 (4.37 ppm) of →4)-*α*-D-GalpA-6-OMe-(1 → (E). This highlighted the presence of a linkage pattern →6)-β-D-Galp-(1 → 4)-*α*-D-GalpA-6-OMe-(1 → (I → E) (45). Similarly, in the NOESY spectrum, the quaternary proton (4.87 ppm) of →4)-*α*-D-GalpA-6-OMe-(1 → (E) showed correlated signals with its own H4 (4.37 ppm). This suggested the presence of a linkage pattern →4)-*α*-D-GalpA-6-OMe-(1 → →4)-*α*-D-GalpA-6-OMe-(1 → (E → E).

##### 3.2.5.4 Comprehensive analysis of possible branching structures

Analysis of possible structure for branch 1: In the HMBC spectrum, the quaternary proton (5.14 ppm) of the glycosidic linkage →2,5)-*α*-L-Araf-(1 → (B) showed correlated signals with its C5 (68.27 ppm) and →5)-*α*-L-Araf-(1 → (D). This indicated the existence of a linkage pattern →2,5)-*α*-L-Araf-(1 → 5)-α-L-Araf-(1 → (B → D). The quaternary proton (5.01 ppm) of the glycosidic linkage →5)-*α*-L-Araf-(1 → (D) showed correlated signals with its C5 (68.27 ppm) and →5)-α-L-Araf-(1 → (D), also suggested the presence of a linkage pattern →5)-*α*-L-Araf-(1 → 5)-α-L-Araf-(1 → (D → D). Similarly, the quaternary proton (5.17 ppm) of the glycosidic linkage α-L-Araf-(1 → (A) showed correlated signals with the C3 (85.15 ppm) of →3,5)-α-L-Araf-(1 → (C), indicating the linkage pattern α-L-Araf-(1 → 3,5)-α-L-Araf-(1 → (A → C). The quaternary proton (5.01 ppm) of the glycosidic linkage →5)-α-L-Araf-(1 → (D) showed correlated signals with the C5 (67.79 ppm) of →3,5)-α-L-Araf-(1 → (C), suggesting the linkage pattern →5)-*α*-L-Araf-(1 → 3,5)-α-L-Araf-(1 → (D → C). The quaternary proton (5.11 ppm) of the glycosidic linkage →3,5)-*α*-L-Araf-(1 → (C) showed correlated signals with the C5 (68.25 ppm) of →2,5)-α-L-Araf-(1 → (B), indicating the linkage pattern →3,5)-α-L-Araf-(1 → 2,5)-α-L-Araf-(1 → (C → B). The quaternary proton (5.17 ppm) of the glycosidic linkage α-L-Araf-(1 → (A) showed correlated signals with the C2 (88.5 ppm) of →2,5)-α-L-Araf-(1 → (B), suggesting the linkage pattern *α*-L-Araf-(1 → 2,5)-α-L-Araf-(1 → (A → B). The quaternary carbon (5.01 ppm) and hydrogen of the glycosidic linkage →5)-α-L-Araf-(1 → (D) showed correlated signals with the H3C3 (81.5 ppm) of →3,6)-*β*-D-Galp-(1 → (G), indicating the linkage pattern →5)-α-L-Araf-(1 → 3,6)-β-D-Galp-(1 → (D → G).

Analysis of possible structure for branch 2: In the HMBC spectrum, the quaternary carbon (105.39 ppm) of the glycosidic linkage →3)-β-D-Galp-(1 → (F) showed correlated signals with the H3 (3.68 ppm) of →3,6)-β-D-Galp-(1 → (G), suggesting the presence of a linkage pattern →3)-β-D-Galp-(1 → 3,6)-β-D-Galp-(1 → (F → G).

Analysis of possible structure for branch 3: In the HMBC spectrum, the quaternary proton (5.17 ppm) of the glycosidic linkage α-L-Araf-(1 → (A) showed correlated signals with the C3 (81.5 ppm) of →3,6)-β-D-Galp-(1 → (G), indicating the linkage pattern →5)-α-L-Araf-(1 → 3,6)-β-D-Galp-(1 → (A → G).

The findings derived from the monosaccharide composition analysis, methylation analysis, and one-dimensional and two-dimensional nuclear magnetic resonance (NMR) spectroscopy data indicate that primary chain connection of BDP-I, it was speculated that the predominant glycosidic linkage structure was →4)-α-D-GalpA-6, while the branching fragments were connected to the main chain through the →3,6)-β-D-Galp-(1 → O-3 linkage, as shown in Figure 2H.

### 3.3 The protective effects against H_2_O_2_-induced oxidative stress

#### 3.3.1 Establishment of H_2_O_2_-induced oxidative stress model in RIN-m5F cells

**Figure 3:**
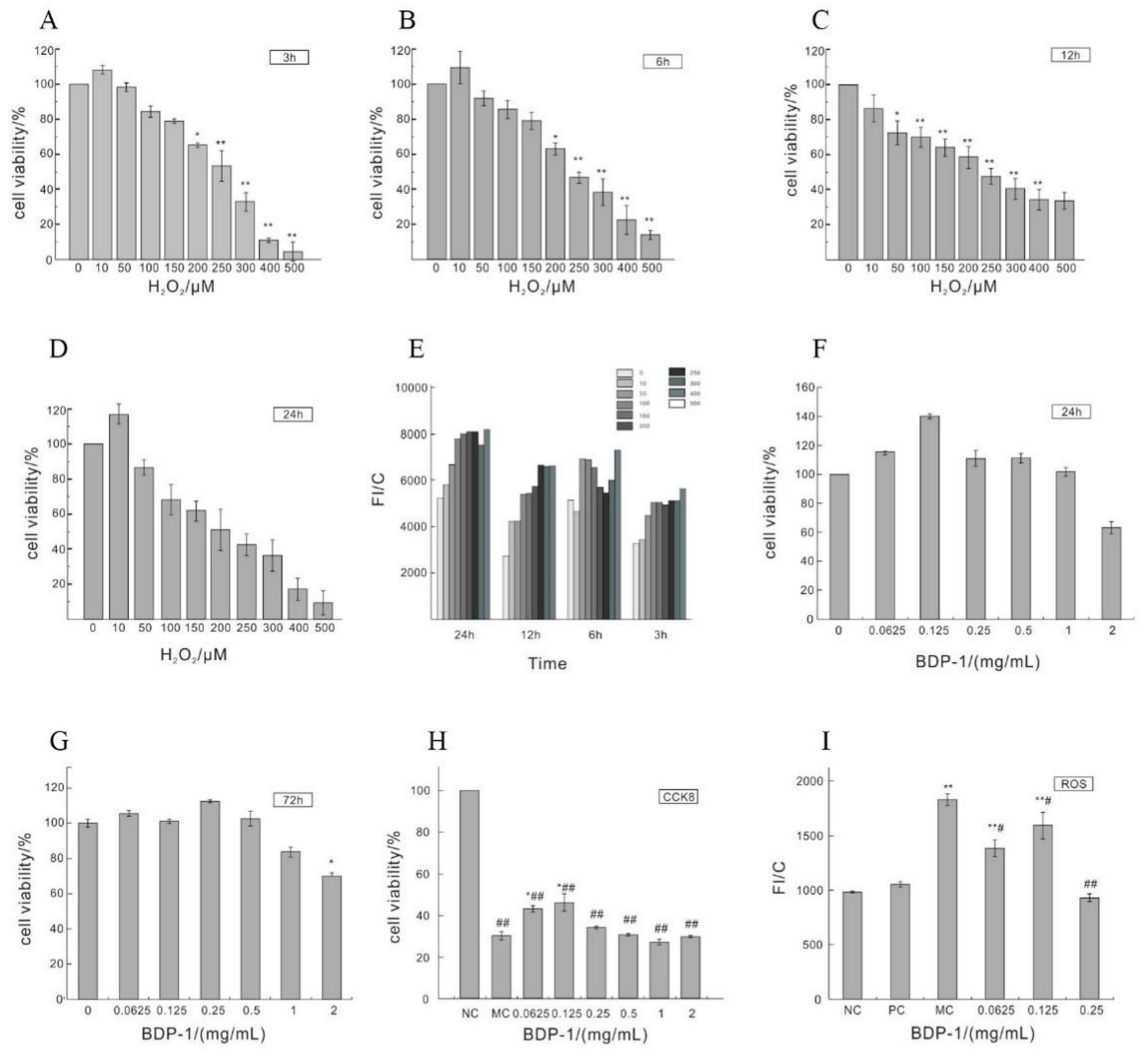
Impact of H_2_O_2_ on the cell viability of RIN-m5F cells. **(A–D)** Depict the influence of H_2_O_2_ induction for 3 h, 6 h, 12 h, and 24 h on cell viability; **(E)** Impact of H_2_O_2_ on ROS levels in RIN-m5F cells; **(F,G)** Effect of different concentrations of BDP-I on cell viability; **(H)** Impact of BDP-I on cell viability in oxidative injury; **(I)** Effect of BDP-I on intracellular ROS levels in oxidative injury. NC, normal control group; PC, positive control group (300 μmol/L *α*-LA); MC, model group (250 μmol/L H_2_O_2_); BDP-I group, BDP-I low-dose group (0.0625 mg/mL), BDP-I medium-dose group (0.125 mg/mL), BDP-I high-dose group (0.25 mg/mL). Different lowercase letters and symbols in the figure indicate significant differences (*p* < 0.05).

The pancreatic is susceptible to damage caused by reactive oxygen species (ROS), which can lead to apoptosis in β-cells and the subsequent development of diabetes (41). Several studies have applied H_2_O_2_ to induce oxidative damage in cells, thereby triggering oxidative injury in β-cells (18, 42). As shown in Figures 3A–D, an increase in the concentration of H_2_O_2_ was associated with a decrease in cell viability, as previously reported by Luo et al.’s findings (18). Compared with the NC, incubating cells with H_2_O_2_ concentrations of 200–500 μmol/L for 3–6 h significantly reduced cell viability (*p < 0.05). Notably, treatment with 250 μmol/L H_2_O_2_ led to a marked reduction in cell viability (*p < 0.01), reaching the half-maximal inhibitory concentration (IC_50_). Figure 3E further demonstrates that the induction of cells with H_2_O_2_ concentrations ranging from 10 to 500 μmol/L resulted in a progressive increase in the ROS levels over time, indicating the influence of H_2_O_2_ on cellular ROS levels. Specifically, the levels of ROS exhibited a rising trend after 3, 6, 12, and 24 h of H_2_O_2_ induction, with a significant elevation observed at a concentration of 100 μmol/L (*p < 0.05 vs NC). Taking into account cell viability, the induction of cells with 250 μmol/L H_2_O_2_ for 3 h was selected as the optimal condition for subsequent experiments.

#### 3.3.2 Cytotoxicity analysis of BDP-I

Figure 3F depicts the impact of BDP-I on cell viability after a 24-h treatment. Cell viability gradually increased within the concentration range of 0.0625 to 0.125 mg/mL, followed by a decline beyond 0.125 mg/mL. Within the range of 0.0625 to 1 mg/mL, there were no statistically significant differences in cell viability between the BDP-I-treated cells at 24 and 72 h compared to the NC (Figures 3F,G). However, at a concentration of 2 mg/mL, cell viability was significantly decreased (*p < 0.05) compared to the NC. These findings confirm that the safe concentration of BDP-I for cells falls within the range of 0.0625 to 1 mg/mL.

#### 3.3.3 Effects of BDP-I on cell proliferation of H_2_O_2_-induced RIN-m5F cell

Figure 3H shows a significant decrease in cell viability after treatment with 250 μmol/L of H_2_O_2_ for 3 h compared to the NC (*p < 0.05). However, when cells were treated with BDP-I at concentrations ranging from 0.0625 to 0.25 mg/mL, cell viability was significantly increased compared to the MC (*p < 0.05). Consequently, it was concluded that BDP-I within this concentration range could enhance cell viability that had been compromised by H_2_O_2_, thereby exerting a protective effect against H_2_O_2_-induced cellular damage.

Figure 3I shows that, following treatment with 250 μmol/L of H_2_O_2_, the MC had significantly higher intracellular ROS levels than the NC (**p < 0.01). In contrast, after 24 h of treatment with 0.0625 to 0.25 mg/mL of BDP-I, there was a significant decrease in the ROS levels of cells compared to the MC (*p < 0.05). These results suggested that H_2_O_2_ induction increased ROS levels in RIN-m5F cells, while BDP-I could reduce these levels after H_2_O_2_-induced damage, thereby inhibiting oxidative stress caused by H_2_O_2_ (18).

#### 3.3.4 Improving cellular oxidative stress of BDP-I

**Figure 4:**
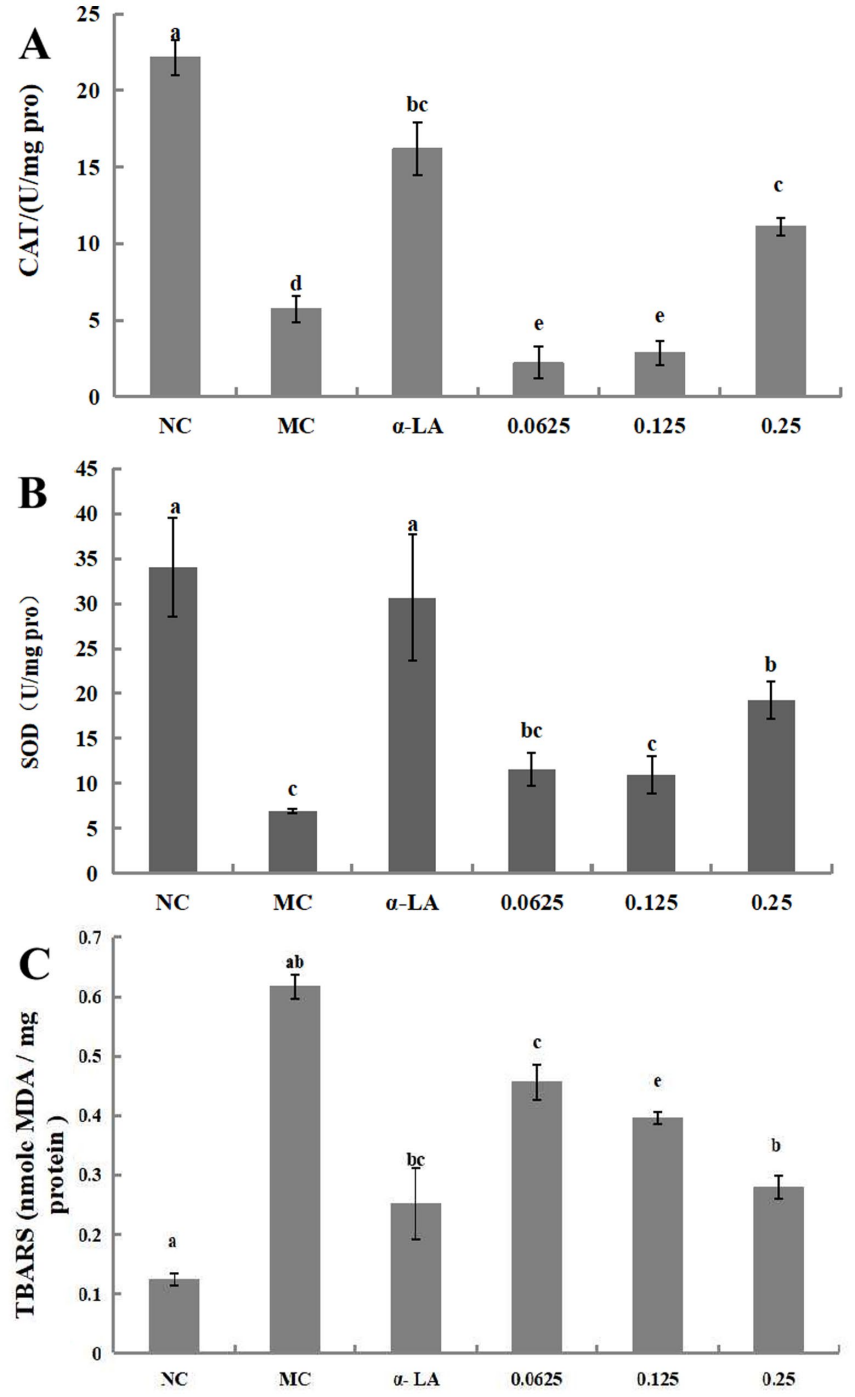
Impact of BDP-I on CAT **(A)**, SOD **(B)**, and TBARS **(C)** levels in RIN-m5F cells. NC, normal control group; α-LA, positive control group (300 μmol/L α-LA); MC, model group (250 μmol/L H_2_O_2_); BDP-I group, BDP-I low-dose group (0.0625 mg/mL), BDP-I medium-dose group (0.125 mg/mL), BDP-I high-dose group (0.25 mg/mL). Different lowercase letters and symbols in the figure indicate significant differences (*p* < 0.05).

The results for SOD and CAT activities as well as the TBARS content in RIN-m5F cells treated with BDP-I, are presented in Figures 4A–C. Following exposure to H_2_O_2_, there was a significant decrease in the activities of SOD and CAT compared to the NC (*p < 0.05). Subsequent treatment with BDP-I resulted in a gradual increase in the activities of these antioxidant enzymes in a dose-dependent relationship. Specifically, at a concentration of 0.25 mg/mL, the SOD activity reached (19.29 ± 2.09) U/mg, while the CAT activity reached (17.45 ± 1.70) U/mg.

Thiobarbituric acid reactive substances (TBARS) assay is extensively utilized to examine the impact of oxidative stress on health and the protective effects of antioxidants. In this study, the influence of BDP-I on lipid peroxidation was assessed by quantifying the lipid peroxidation product, TBARS, as depicted in Figure 4C. The TBARS level in untreated cells was measured at 0.123 ± 0.01 nmol malondialdehyde (MDA), whereas exposure to hydrogen peroxide (H_2_O_2_) significantly elevated TBARS levels to 0.617 ± 0.02 nmol MDA. Conversely, treatment with BDP-I at concentrations of 0.0625, 0.125, and 0.25 mg/mL markedly reduced the elevated TBARS levels induced by H_2_O_2_ (*p* < 0.05), with measurements recorded at 0.456, 0.395, and 0.279 nmol MDA, respectively.

### 3.4 BDP-I regulated metabolomics in H_2_O_2_-induced oxidative stress

#### 3.4.1 QC and multivariate statistical analysis for cell metabolome

The total ion chromatogram (TIC) of QC samples were compared and analyzed. In order to examine the overall metabolic changes in the sample profiles, separate PCA analyses were performed for the MC, NC, and BDP-I-treated group using SIMCA-P software (Supplementary Table S2). The ellipses on the graph subsequently represented the 95% confidence interval. The distinct separation observed between the NC and MC indicates the successful establishment of the oxidative stress cell model (R^2^X = 0.658), with significant alterations in cellular metabolites. The distinction between the polysaccharide group and the MC was less pronounced, whereas other groups displayed notable differences but were not entirely clustered. A supplementary PLS-DA analysis was performed to delve deeper into the disparities and patterns in cellular metabolites between the various groups. Orthogonal Partial Least Squares Discriminant Analysis (OPLS-DA) is an adapted analytical approach for PLS-DA.

This study employed the variable importance in the projection (VIP) values derived from the OPLS-DA model to identify potential biomarkers. Table 3 shows significant differences between the MC and NC groups in positive and negative ion modes, indicating cell metabolic disturbances after oxidative damage. The sample distribution of the BDP-I-treated group approaches that of the NC, suggesting a potential protective effect of BDP-I on oxidatively damaged cells.

**Table 3:** Potential biomarkers and trends in BDP-I intervention for oxidative damage cells.

Differential metabolites names	HMDB ID	FC_M_	FC_B_	MC vs NC	BDP-I vs MC
Mevalonic acid	0059629	0.49	1.02	↓**	↑^###^
Cytidine 5′-monophosphate(CMP)	0000095	0.25	0.55	↓**	↓
DL-serine	0062263	0.3	0.67	↓***	↓^###^
D-glucosamine 1-phosphate	0001109	0.44	0.58	↓*	↓^###^
alpha-D-Galactose-1-phosphate	0000645	0.51	0.92	↓**	↓^###^
Pyruvate	0000243	1.34	0.29	↓	↑^###^
Ile-Pro	0000012	0.16593	1.31	↓**	↑
Isopentenyl pyrophosphate	0001347	0.3	0.83	↓**	↓^###^
DL-tryptophan	0030396	0.16	0.55	↓**	↓^##^
Adenosine 3′,5′-cyclic phosphate (cAMP)	0000058	0.75	0.66	↓	↓^#^
Guanosine 5′-monophosphate (GMP)	0001397	0.5	0.72	↓**	↓^##^
Xanthine	0000292	0.41	0.43	↓	↓
Asparagine	0000168	0.09	0.36	↓**	↓^##^
Gdp-l-fucose	0001095	0.33	0.93	↓**	↓^##^
Uracil	0000300	0.21	0.86	↓**	↓^##^
Cytidine monophosphate-n-acetylneuraminic acid	0001176	0.13	0.36	↓**	↓^##^
D-ribose 1-phosphate	0001489	0.36	0.41	↓*	↓
Udp-xylose		0.35	0.56	↓**	↓^##^
Thymine	0000262	0.35	0.94	↓**	↓
N-acetylneuraminate	0000230	0.41	0.6	↓	↓^###^
His-Lys	0000133	0.3	0.71	↓**	↓
N-acetyl-d-glucosamine-6-phosphate	0001062	0.3	0.57	↓**	↓^##^
Aspartic acid	0000191	0.23	0.42	↓**	↓^##^
Udp-galactose	0000302	0.16	0.65	↓**	↓^##^
Xanthosine	0000299	0.47	0.31	↓	↓
Deoxythymidine 5′-phosphate (dTMP)	0001227	0.32	0.79	↓	↓
Udp-n-acetylglucosamine	0000290	0.15	0.21	↓**	↓^#^
Pantetheine	0003426	0.58	0.34	↓*	↓^#^
Deoxyinosine	0000071	0.27	0.71	↓**	↓
D-gluconate	0000625	0.35	0.43	↓*	↓^#^
His-ser	0000273	0.31	1.53	↓**	↑
beta.-nicotinamide adenine dinucleotide(NAD)	0000902	0.35	0.63	↓**	↓^#^
D-glucose 6-phosphate		0.68	0.93	↓**	↓^#^
L-Phenylalanine	0000159	0.57	0.74	↑	↓^#^
Fumarate	0000134	0.25	0.78	↓**	↓^#^
D-ribulose 1,5-bisphosphate		0.47	0.54	↓*	↓^#^
Uridine 5′-monophosphate	0000288	0.17	0.54	↓**	↓
N-acetyl-L-aspartic acid	0000812	0.34	0.61	↓*	↓^#^
L-Glutamate	0000148	1.23	0.5	↓	↑^#^
Alanine	0000161	0.27	0.49	↑*	↓^#^
Thiamine pyrophosphate (tpp)	0001372	0.39	0.61	↓*	↓^#^
Acetyl coenzyme a	0001206	0.21	0.56	↓**	↓^#^
Diadenosine triphosphate		0.57	0.84	↓*	↓
Inosine 5′-monophosphate	0000175	0.2	1.39	↓**	↑
Alpha-ketoisovaleric acid	0000019	1.95	0.45	↑	↓^#^
Uridine 5′-diphosphoglucose	0000286	0.58	0.91	↓	↓^#^

↑ Indicates upregulation; ↓ indicates downregulation. *Significant changes in MC vs NC (**p < 0.01; *p < 0.05). #Significant changes in BDP-I vs MC (^##^*p* < 0.01; ^#^*p* < 0.05). NC, normal control group; PC, positive control group; MC, model group; BDP-I: BDP-I high-dose group (0.25 mg/mL).

#### 3.4.2 Differential metabolite analysis

**Figure 5:**
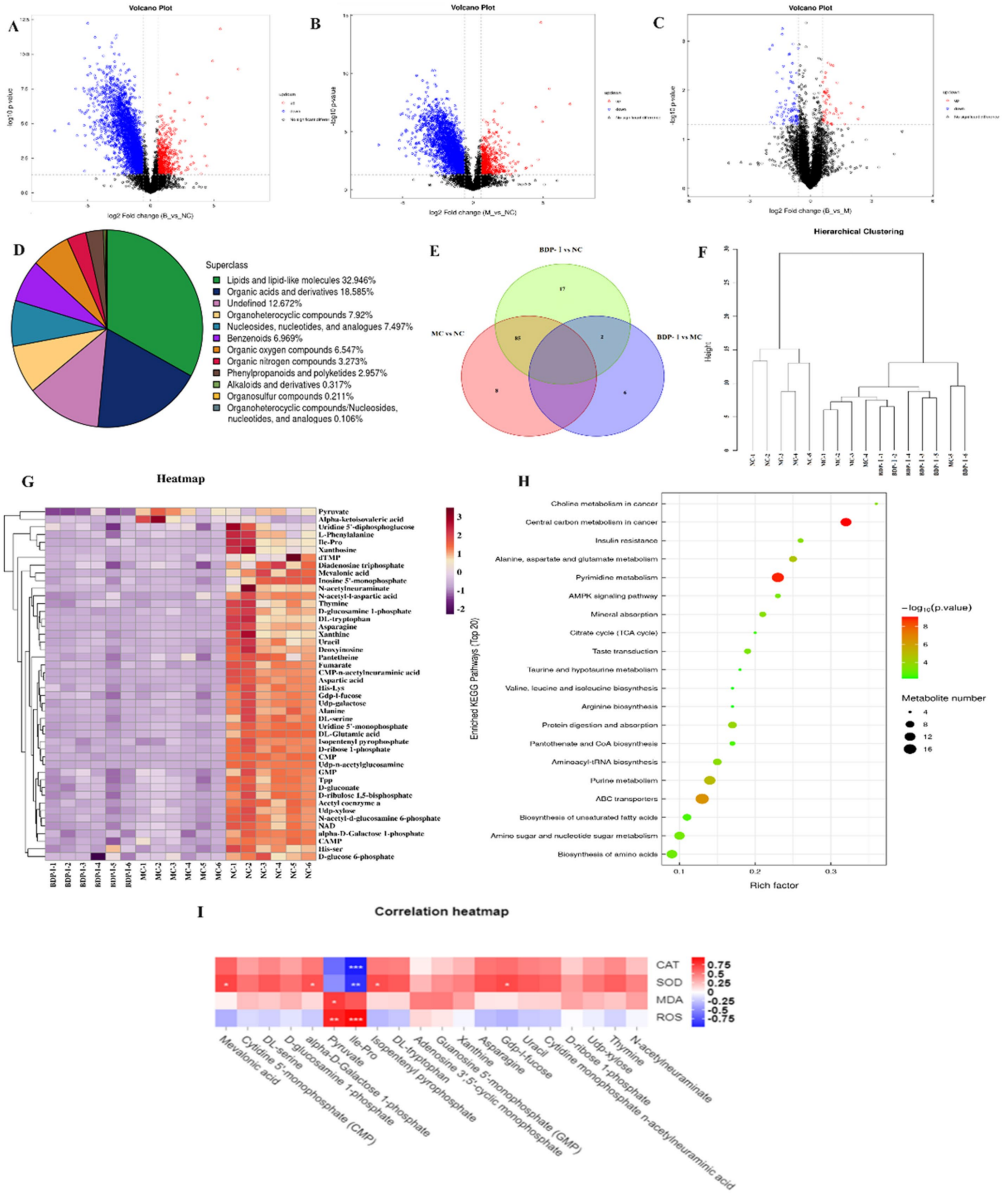
**(A–D)** Volcano plot of differential metabolites in negative ion mode; **(E)** OTU relationships between different groups; **(F)** Hierarchical clustering dendrogram; **(G)** Hierarchical clustering heatmap; **(H)** Bubble chart of KEGG pathway enrichment; **(I)** Correlation between metabolites and oxidative stress phenotype.

A comprehensive analysis of metabolites was conducted, identifying 947 metabolites in both positive and negative ion modes. Specifically, 501 metabolites were identified in positive ion mode, while 446 metabolites were identified in the negative one. These metabolites were subsequently categorized and quantified based on information on their chemical classification. The distribution of metabolites within each class is visually represented in Figure 5A. Furthermore, the OPLS-DA model analysis identified significant differential metabolites, with VIP > 1 and *p < 0.05* selected at thresholds (Supplementary Figure S1). In addition, the results were compared with the results from the HMDB and KEGG databases. In the negative ion mode, 169 differential metabolites were identified. Of these, lipids and lipid-like molecules accounted for the largest proportion at 32.946%. Organic acids and derivatives followed at 18.585%, while heterocyclic compounds comprised 7.92% of the identified metabolites. Additionally, nucleosides, nucleotides, and similar compounds constituted 7.497%, benzene compounds accounted for 6.969%, and organic oxygen compounds represented 6.547%. These metabolites were primarily concentrated in glycerophospholipids, nucleotides, carboxylic acids and derivatives, fatty acyls, and organic oxygen compounds.

Following the completion of univariate analysis, a differential analysis was conducted on all identified metabolites (including unidentified ones), encompassing both positive and negative ion modes. A further screening process was then undertaken to identify differential metabolites that met the criteria of a fold change (FC) of >1.5 or <0.67 and a *p*-value <0.05. Using a volcano plot allowed the expression patterns of differential metabolites in the negative ion mode to be visually presented (Figures 5B–D). Within this plot, each point corresponded to a specific metabolite, with the size of the point indicating the Variable Importance in Projection (VIP) value. Upregulated metabolites are depicted in red, while downregulated ones are resented in blue. However, metabolites that did not reach statistical significance are displayed in black. Compared to the NC, the MC exhibited an increase in 17 metabolites and a decrease in 44 metabolites. Similarly, the BDP-I group displayed 7 upregulated metabolites and 15 downregulated ones compared to the MC group (Figure 5E).

#### 3.4.3 Identification of potential biomarkers

The results presented in Table 3 illustrate potential biomarkers and trends after BDP-I treatment in cells displaying oxidative damage. Notably, BDP-I intervention had a significant impact on metabolic pathways. As indicated in Table 3, a total of 46 metabolites were found to be associated with T2DM, including 9 carboxylic acids and derivatives, 8 organic oxides, 10 pyrimidine nucleotides, 3 fatty acyls as well as 2 keto acids and derivatives: Gdp-l-fucose, 3 pyrazines, 1 imidazole compound (xanthine), and other compound groups such as amino acid-lysine, adenosine triphosphate, isopentyl pyrophosphate, DL-tryptophan, and β-nicotinamide adenine dinucleotide. After the implementation of the BDP-I intervention, the concentrations of phenylalanine, L-glutamic acid, pyruvic acid, *α*-ketoisovaleric acid, methylhydroxyvalerate, isoleucine-valine, 5′-deoxythymidine monophosphate (dTMP) and guanosine-5′-monophosphate were reversed, suggesting their potential as biomarkers for BDP-I intervention in pancreatic cells affected by oxidative damage.

Cluster analysis can provide a comprehensive visualization of the associations between samples while highlighting the distinct expression patterns of metabolites across different samples. Hierarchical cluster analysis was performed on samples from each group, and the resulting cluster tree is shown in Figure 5F, where samples clustered in the same branch display higher similarity. The NC was clearly segregated from the other two groups in this case. At the same time, the NC samples demonstrated cohesive clustering, indicating a high degree of similarity between them. This observation suggested the effective establishment of the cellular oxidative stress model at the metabolomic level. Figure 5G presents a cluster heatmap, revealing that pyruvic acid was significantly upregulated in the MC group but downregulated in the BDP-I group. Conversely, α-ketoisovaleric acid was downregulated in the NC and BDP-I-treated groups while upregulated in the MC.

#### 3.4.4 Metabolic pathway analysis

Figure 5H illustrates the 15 pathways found to be associated with T2DM (*p < 0.05*), namely the central carbon metabolism in cancer, insulin resistance, alanine, aspartate and glutamate metabolism, insulin secretion, tricarboxylic acid (TCA) cycle, cGMP-PKG signaling pathway, taurine, and hypotaurine metabolism, pantothenate and CoA biosynthesis, insulin signaling pathway, rust disease, AMPK signaling pathway, oxidative phosphorylation, amino sugar and nucleotide sugar metabolism and carbon metabolism. These results suggest that by modulating these pathways, BDP-I primarily affects the oxidative damage to pancreatic β-cells.

Pancreatic β-cells are essential in maintaining equilibrium in Glc metabolism by facilitating insulin secretion (43). However, oxidative stress triggers the apoptosis of β-cells, leading to diminished insulin secretion. Research has demonstrated that in a T2DM animal model, antioxidants could potentially alleviate hyperglycemia, thereby regulating the typical expression of the insulin promoter PDX-1 and transcription factor MafA (46). Consequently, exploring new diabetes treatment and pathogenesis through the lens of oxidative stress holds promise for identifying novel active constituents or therapeutic strategies for efficacious disease management.

Based on previous investigations on cellular phenotypes, it was observed that BDP-I exhibited a good antioxidant effect by enhancing the viability of cells damaged by oxidative stress and augmenting the performance of antioxidant enzymes. Metabolomic analyses provided additional evidence that BDP-I can counteract the metabolic disturbances induced by oxidative damage by influencing 46 potential biomarkers. Glc is the primary energy source of glycolysis and the TCA cycle, universally present metabolic pathways in aerobic organisms. Succinic acid plays a key role in sugar metabolism, promoting ATP generation, exhibiting antioxidant stress effects, increasing tissue levels of GSH and SOD, reducing MDA content, inhibiting the expression of inflammatory factors and protecting cells from excessive apoptosis (47). In the present study, a reduction in succinic acid content was observed in the MC. However, following BDP-I intervention, a significant increase in succinic acid levels was observed. Given the involvement of succinic acid in multiple metabolic pathways, including the TCA cycle, these findings suggest that BDP-I may exert its therapeutic effects on oxidative stress by intervening in the metabolism of oxidatively damaged cells through diverse pathways and targets.

Additionally, alanine, which involved in various metabolic processes and is a crucial energy source, plays a significant role in amino acid metabolism. Research has demonstrated that in diabetic patients, plasma alanine levels tend to be elevated (48). However, this which can effectively be reduced by BDP-I, improving amino acid metabolism disorders. The MC exhibited notable reductions in the levels of amino acids such as serine and alanine, while the levels of phenylalanine and L-glutamine displayed the opposite effects. Phenylalanine, an aromatic amino acid, can be a predictive factor for diabetes risk and is positively associated with the risk of T2DM (49). L-glutamine, a non-essential amino acid abundantly present in the body, acts as a substrate for bodily metabolism and can enhance insulin secretion (50). Therefore, BDP-I may improve T2DM by regulating phenylalanine and L-glutamic acid levels to stimulate insulin secretion.

The metabolism of nucleotides in cells experiencing oxidative damage display alterations, especially with regards to a notable reduction in the concentrations of the four pyrimidine nucleotides (hydroxyethyl glucoside, Udp-N-acetylglucosamine, Udp-galactose, and cytidine-N-acetylneuraminic acid) and one purine nucleotide (Gdp-l-fucose). Purine metabolism is vital in preserving the equilibrium of mitochondrial oxidative stress within the organism, as it plays a pivotal function in providing energy, metabolic control, and coenzyme composition (51). The high levels of pyrimidine nucleotides in the MC led to oxidative stress in renal cells. The findings mentioned above suggest a strong correlation between the antioxidant mechanism of BDP-I and various metabolic processes, including alanine, aspartate, and glutamate metabolism, insulin secretion, the TCA cycle, the cGMP-PKG signaling pathway, taurine and hypotaurine metabolism, pantothenate and coenzyme A biosynthesis, the glucagon signaling pathway, rust disease, the AMPK signaling pathway, oxidative phosphorylation, amino sugar and nucleotide sugar metabolism, and disruption of carbon metabolism.

#### 3.4.5 Correlation analysis between phenotypic changes and differential metabolites with BDP-I intervention

To investigate the association between metabolites and cellular oxidative stress, Spearman correlation analysis was used to evaluate the relationship between crucial differential metabolites and indicators related to oxidative stress (Figure 5I). The heatmap illustrates positive correlations in red and negative correlations in blue, with the intensity of color indicating the magnitude of the correlation. Specifically, isoleucine-proline and pyruvic acid exhibited a positive correlation with reactive oxygen species (ROS) and malondialdehyde (MDA) while displaying a negative correlation with superoxide dismutase (SOD) and catalase (CAT). These findings suggest that an elevation in isoleucine-proline and pyruvic acid levels may potentially enhance cellular oxidative stress. In addition, methylhydroxybutyrate, alpha-D-mannose-1-phosphate, isocitric acid isobutyl ester, Gdp-l-fucose showed a positive correlation with SOD but a negative correlation with ROS, indicating that these metabolites could have been associated with enhanced intracellular antioxidant enzyme activity and inhibited oxidative stress.

### 3.5 The potential mechanism of protective effects against H_2_O_2_-induced oxidative stress

After identifying the metabolic pathways in which significantly different metabolites were present, they were matched to the KEGG database. Subsequent enrichment analysis was conducted on the annotated results to identify metabolic pathways associated with T2DM. Supplementary Figure S2 displays the TCA cycle and oxidative phosphorylation pathway, highlighting the upregulation of acetoacetate, usnic acid, and acetyl-CoA through red circles. These metabolite changes are also linked to lipid metabolism, glycolysis, amino acid metabolism, and the oxidative phosphorylation pathway. The mitochondrial oxidative phosphorylation pathway also plays a vital role in producing cellular energy by supplying ATP to the organism. This process occurs on the inner mitochondrial membrane of eukaryotic cells and involves the participation of complexes I to V. The malfunction of complex I (NADH dehydrogenase) has been associated with metabolic disorders in oxidative stress. Complex II (succinate dehydrogenase) serves as an enzyme in both the TCA cycle and the electron transport chain, whereas complex III (cytochrome C oxidoreductase) functions as a dimer responsible for catalyzing ubiquinone oxidation and cytochrome C reduction. Complex IV, also known as cytochrome c oxidase, represents the terminal protein complex within the electron transport chain (52). On the other hand, Complex V, or ATP synthase, serves as the ultimate enzyme within the oxidative phosphorylation pathway, and modulation of its function can impact mitochondrial permeability. The increased NAD + and gentisic acid levels imply an elevation in complexes I and II abundance. Research has demonstrated that increased proteins associated with mitochondrial complexes I and IV enhance mitochondrial function. Therefore, BDP-I may improve antioxidant activity by upregulating the expression of NAD + and gentisic acid metabolites, increasing the expression of related proteins, and suppressing mitochondrial oxidative stress (Supplementary Figure S2).

### 3.6 Validation of cellular function in BDP-I intervention for oxidative damage of pancreatic islet cells

**Figure 6:**
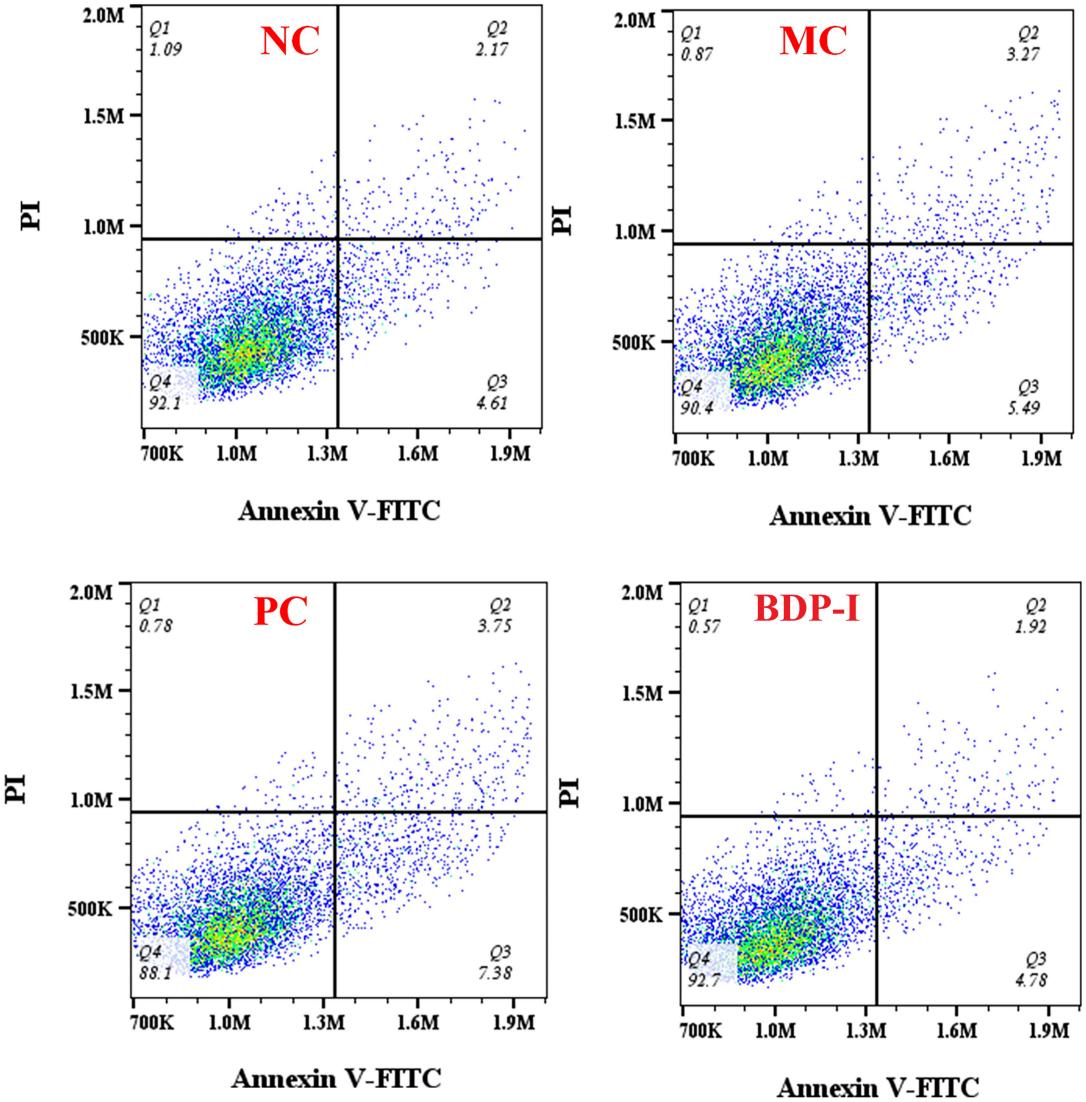
Changes in apoptosis rate of pancreatic islet cells in different treatment groups. NC, normal control group; PC, positive control group; MC, model group; BDP-I, BDP-I high-dose group (0.25 mg/mL).

Apoptosis rate was measured by flow cytometry (Figure 6; Table 4). The impact of BDP-I on the apoptosis of pancreatic islet cells induced by hydrogen peroxide is illustrated in the figure. The findings indicate that following exposure to H_2_O_2_, the total apoptosis indices of the MC group cells were as follows: 9.9%. Notably, the apoptosis index was significantly elevated in the NC group. However, following intervention in both the PC group and the BDP-I group, the apoptosis index markedly decreased, with the apoptosis rate being significantly lower than that observed in the MC group (*p < 0.05*). The H_2_O_2_-induced cell damage model leads to a substantial increase in the rate of cell apoptosis. Intervention with BDP-I and the positive control drug (*α*-LA) effectively mitigates the rate of cell apoptosis and BDP-I increased the rate of living cells up to 90.53 ± 0.87%, thereby offering further protection against H_2_O_2_-induced cellular damage.

**Table 4:** The rate of RIN-m5F cells apoptosis of different groups.

Group	Q1 (Dead cells)%	Q2 (Late apoptotic cells)%	Q3 (Early apoptotic cells)%	Q4 (Living cell)%
NC	1.03 ± 0.05	2.28 ± 0.09^b^	4.75 ± 0.09^b^	91.97 ± 0.09^a^
MC	0.76 ± 0.14	3.30 ± 0.32^ab^	6.60 ± 0.30^a^	89.33 ± 0.33^bc^
PC	0.93 ± 0.19	4.01 ± 0.4^a^	7.41 ± 0.64^a^	87.63 ± 0.95^c^
BDP-I	0.7 ± 0.05	2.76 ± 0.41^b^	5.99 ± 0.43^ab^	90.53 ± 0.87^ab^

Different letters on the same shoulder label indicate significant differences (*p* < 0.05). NC, normal control group; PC, positive control group; MC, model group; BDP-I, BDP-I high-dose group (0.25 mg/mL).

### 3.7 Validation of potential regulatory gene expression in BDP-I intervention for oxidative damage in pancreatic islet cells

**Figure 7:**
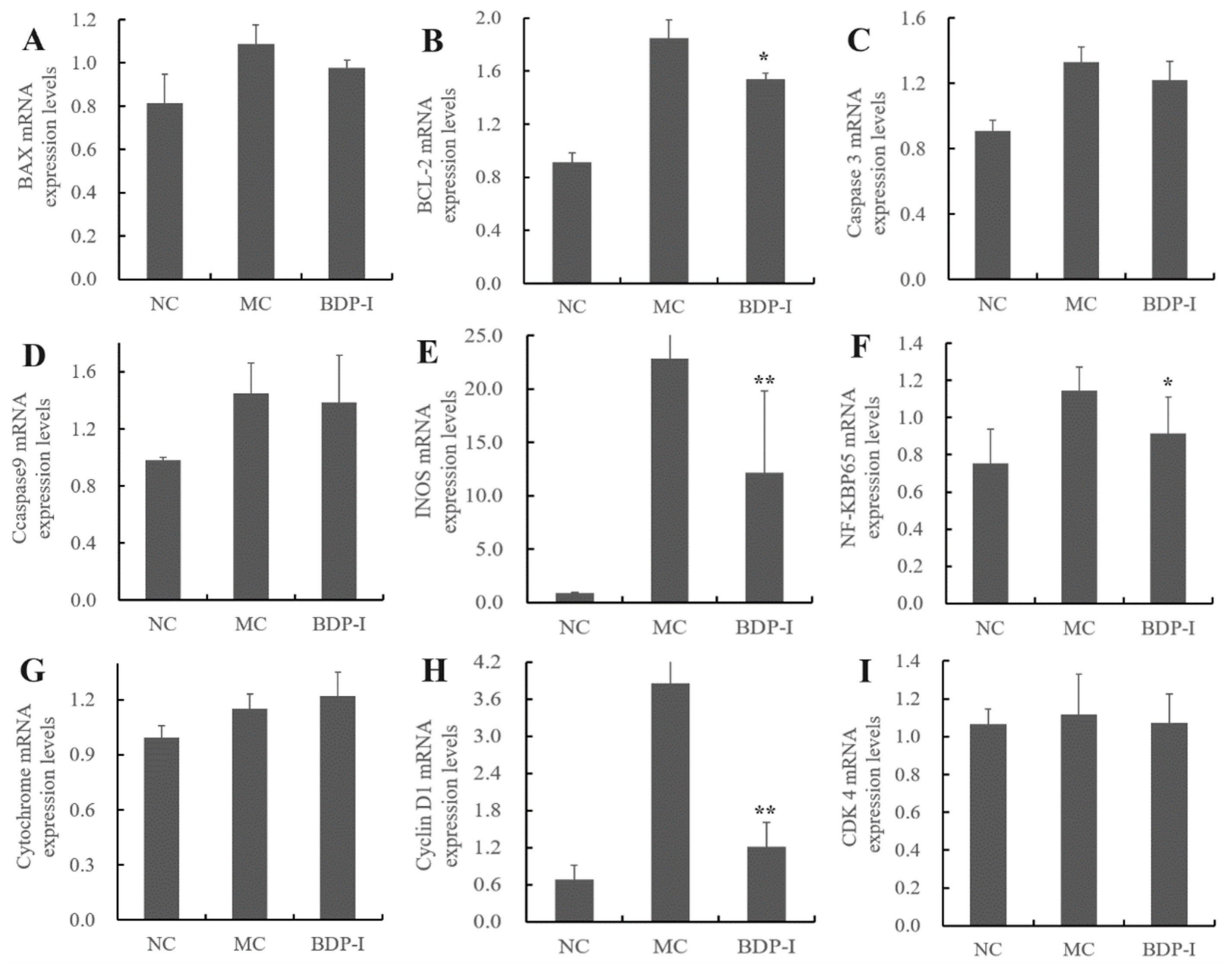
The impact of BDP-I intervention on the regulation of mRNA expression levels in oxidatively damaged pancreatic islet cells. The mRNA expression levels of **(A)** BAX; **(B)** BCL-2; **(C)** Caspase 3; **(D)** Caspase 9; **(E)** INOS; **(F)** NF-KBP65; **(G)** Cytochrome; **(H)** Cyclin D1; and **(I)** CDK 4 were measured by qRT-PCR. NC, normal control group; MC, model group; BDP-I, BDP-I high-dose group (0.25 mg/mL), *p < 0.05, and **p < 0.01 vs. MC group.

Based on the analysis of cellular metabolomics and KEGG pathways as well as through reference to previous studies (18, 41, 53, 54), qRT-PCR analysis was found to assess the mRNA expression levels of cyclin D1, CDK 4, Bax/Bcl-2, caspase 3, caspase 9, Cyt C, iNOS, and NF-κB. As depicted in Figure 7, the MC exhibited increased expression levels of apoptosis-related genes (Bax, Bcl-2, caspase 3, caspase 9, Cyt C) compared to the NC. However, following BDP-I intervention, the expression of Bax, Bcl-2, caspase 3, and caspase 9 decreased. In particular, Bcl-2, an anti-apoptotic gene, and its homologous gene Bax, are involved in promoting cell apoptosis (18). The caspase family, which consists of cysteine-aspartic acid proteases, is a major inducer of cell apoptosis (55). The gene expression levels of Bax, caspase 3, caspase 9, and Cyt C increased in the MC, but decreased after BDP-I intervention. This suggests that BDP-I may inhibit cell apoptosis by regulating the expression of apoptotic genes. In this experiment, the induction of H_2_O_2_ resulted in a significant increase in ROS levels in the MC, leading to an upregulation of iNOS.

Oxygen-derived species, functioning as second messengers, can activate the nuclear transcription factor NF-κB and play a role in inflammation (46). The MC exhibited elevated levels of oxidative stress due to the upregulation of iNOS gene expression and NF-κB proteins. However, the expression levels demonstrated a decreasing pattern following the intervention of BDP-I. The results obtained from qRT-PCR analysis provided evidence that BDP-I intervention could effectively safeguard pancreatic islet cells against oxidative stress-induced damage and interfere with the apoptosis of pancreatic islet cells by regulating the genes involved in the apoptotic cycle (cyclin D1, CDK 4) and apoptosis-related genes (Bax/Bcl-2, caspase 3, caspase 9, Cyt C), and mitochondria-related genes (iNOS, NF-κB).

Reactive oxygen species (ROS) play a crucial role in oxidative stress and represent an unavoidable consequence of metabolic processes. The increase in ROS levels disturbs the balance between oxidants and antioxidants, resulting in a condition called oxidative stress. Under normal physiological circumstances, the pancreas’s limited antioxidant capacity, makes it vulnerable to oxidative attacks (47). Prolonged exposure of pancreatic β-cells to high concentrations of free fatty acids or Glc reduces the amount of intracellular antioxidant enzymes such as SOD and GSH-Px, thereby triggering apoptosis in pancreatic β-cells (49). Oxidative stress exerts a multifaceted influence on pancreatic islet injury, impacting insulin synthesis, secretion, and signaling pathways. Currently, numerous studies investigate the pathogenesis of oxidative stress-induced diabetes, with particular attention to the mechanisms of oxidative damage and effective targets within signaling pathways, which have emerged as focal points in this research area. Consequently, a comprehensive examination of oxidative stress pathways is of paramount importance for elucidating the mechanisms of damage and advancing therapeutic strategies for diabetes.

The preliminary investigation conducted by the research group identified BDP as a compound exhibiting significant physiological activities, including potent biological functions such as hypoglycemic and antioxidant effects. The biological activity of polysaccharides is intricately linked to their structural characteristics. Consequently, this study employed spectral, chromatographic, and mass spectrometry techniques to isolate and purify crude polysaccharides derived from *Berberis dasystachya* M, with the objective of elucidating the detailed structure of uniform polysaccharides from this species. The overarching goal is to elucidate the chemical structure and active substance basis of the efficacious active components found in medicinal and edible berry resources from the Qinghai-Tibet Plateau. The results in this work indicate that treatment with BDP-I can enhance the activity of antioxidant enzymes within cells, thereby alleviating pancreatic cell damage caused by H_2_O_2_. The antioxidant activity of plant polysaccharides is significantly influenced by factors such as monosaccharide composition, molecular weight, and the degree and size of branching points. BDP-I demonstrates exceptionally strong antioxidant activity, which we hypothesize may be attributed to variations in carbohydrate structure, monosaccharide composition, glycosidic bond configuration, and other molecular components. Preliminary analyses reveal that BDP-I is composed of Ara: Gal: Glu: GalA in a ratio of 108:99:89:704, with galacturonic acid identified as the predominant monosaccharide. Previous research by Ji et al. has shown that three homogeneous polysaccharides isolated from red dates exhibit increased antioxidant activity with higher galacturonic acid content (10). We propose that the substantial presence of galacturonic acid in BDP-I constitutes the fundamental basis for its *in vitro* antioxidant properties.

Our prior research has demonstrated that the molecular weight of the purified crude polysaccharide is 24.3 kDa (4), whereas BDP-1 is characterized as a purified polysaccharide with a lower molecular weight of 3.947 kDa. Consequently, it is anticipated that BDP-1 exhibits higher activity compared to the unpurified polysaccharide with a higher molecular weight. Polysaccharides with low molecular weight generally exhibit enhanced antioxidant capacity. This enhancement is attributed to the increased solubility and bioavailability associated with lower molecular weights, which subsequently augments their biological activity (56). Notably, specific low molecular weight polysaccharides demonstrate significant efficacy in scavenging free radicals, thereby presenting extensive potential applications in the domains of food and medicine (57).

Monosaccharides are linked by glycosidic bonds, and the nature and configuration of these bonds significantly influence the biological activity and bioavailability of polysaccharides (58). According to the findings of Baruah et al. (59), β-glucans, a crucial class of polysaccharides, demonstrate notable biological activity in immune modulation attributable to the presence of β-(1 → 3) and β-(1 → 4) glycosidic bonds within their structure. These polysaccharides have the capacity to activate immune cells and initiate immune responses through their interaction with specific receptors. The impact of *Lycium barbarum* polysaccharides from various regions on enhancing macrophage function differs, potentially due to the chemical characteristics of their glycosidic bonds. Nonetheless, research indicates that certain polysaccharides exhibit resistance to enzymatic degradation during digestion, attributable to their intricate glycosidic bond structures, which consequently influences their bioavailability. For instance, sulfated fucoidan found in seaweed demonstrates significant biological activity owing to its distinctive glycosidic bond configuration; however, its digestion and absorption within the body remain constrained (60). Simultaneously, studies have demonstrated that the biological activity of Porphyra haitanensis polysaccharide (CPH) on RAW264.7 cells exposed to H_2_O_2_ is comparatively low, thereby limiting its applicability. Nonetheless, when CPH is subjected to enzymatic hydrolysis to produce DCPH polysaccharides, the resulting compounds exhibit enhanced proliferation, phagocytosis, and nitric oxide (NO) secretion activities relative to CPH (61).

However, the link between polysaccharides’ activity and their chemical structure is not fully understood, hindering deeper research and the development of these resources. Future research should further investigate the interactions among these structure–activity relationship to enhance the effective utilization of polysaccharides as antioxidants and functional food ingredients.

Consequently, this study performed non-targeted metabolomics through UHPLC–MS/MS technology to investigate the mechanisms that would allow elucidation of the impacts of BDP-I on oxidative damage within pancreatic islet cells. The findings revealed disruptions in cellular metabolism during oxidative stress, with BDP-I intervention inducing modifications in metabolic pathways. By modulating 46 potential biomarkers, BDP-I can influence pathways associated with alanine, aspartate, and glutamate metabolism, insulin secretion, TCA cycle, cGMP-PKG signaling pathway, nucleotide metabolism, and carbon metabolism. Moreover, the BDP-I-based treatment of pancreatic islet cells involved in the modulation of apoptosis rate and apoptosis-related genes within the cell cycle (cyclin D1, CDK 4), especially the apoptotic pathway genes (Bax/Bcl-2, caspase 3, caspase 9, Cyt C), and genes linked to mitochondrial damage (iNOS, NF-κB), thereby intervening in the apoptosis of pancreatic islet cells.

## 4 Conclusion

In this study, BDP-I was obtained through column separation and purification, BDP-I contained 10 different glycosidic linkage type: →5)-*α*-L-Araf-(1→, →5)-α-L-Araf-(1→, →3,5)-α-L-Araf-(1→, →2,5)-α-L-Araf-(1→, →3,6)-β-D-Galp-(1→, →6)-β-D-Galp-(1→, →3)-β-D-Galp-(1→, β-D-Galp-(1→, →4)-β-D-Manp-(1→, →4)-α-D-GalAp-(1→. The predominant linkage in this polysaccharide was inferred to be connected by →6)-β-D-Galp-(1 → glycosidic bonds. The pancreatic islet cell oxidative damage model was established by H_2_O_2_ induction in RIN-m5F cells, and it was found that BDP-I showed a significant increase in the activities of SOD and CAT compared to the MC (*p < 0.05). Non-targeted metabolomics results revealed that achieved this by modulating 46 potential biomarkers(*p < 0.05) and BDP-I increased the rate of living cells up to 90.53 ± 0.87%, BDP-I intervening in pancreatic islet cell apoptosis through apoptotic cycle-related genes (cyclin D1, CDK 4), apoptotic pathway-related genes (Bax/Bcl-2, caspase 3, caspase 9, Cyt C), and mitochondrial damage-related genes (iNOS, NF-κB). This study systematically revealed the molecular mechanisms of BDP-I intervention in pancreatic islet cell apoptosis, providing theoretical foundations for effectively using yellow thorn berries from the Qinghai-Tibet Plateau. Nonetheless, our study was limited to examining the structure of BDP-I and it’s *in vitro* activity in inhibiting apoptosis in pancreatic cells. Further research is required to elucidate its precise structure–activity relationship.

## Data Availability

The original contributions presented in the study are included in the article/Supplementary material, further inquiries can be directed to the corresponding author.
