# Supplementary material for: Structural characterization and protective effects of polysaccharides isolated from Berberis dasystachya M. fruits on H2O2-induced oxidative damage in RINm5F pancreatic β-cells
Source: Front Nutr. 2025 Aug 6;12:1643051. doi: 10.3389/fnut.2025.1643051 (PMC12364817; doi:10.3389/fnut.2025.1643051)
Supplement: Supplementary file 1 [file Data_Sheet_1.docx]

Supplementary Material

# Supplementary Figures and Tables

## Supplementary Tables

**Supplementary Table S1** Primers of RT-PCR analysis for genes

| Target Gene | Primer sequence (5ʹ-3ʹ) | fragment length（bp） |
| --- | --- | --- |
| BCL-2-F | TTGTGGCCTTCTTTGAGTTCG | 151 |
| BCL-2-R | GCATCCCAGCCTCCGTTAT |  |
| Bax-F | GGGTGGTTGCCCTTTTCTACTT | 104 |
| Bax-R | GAAGTCCAGTGTCCAGCCCAT |  |
| Cyclin D1-F | GATGCTAGAGGTCTGCGAGGAG | 212 |
| Cyclin D1-R | GGCGGATAGAGTTGTCAGTGTAGAT |  |
| iNOS-F | CTACTACTACCAGATCGAGCCCTG | 197 |
| iNOS-R | CTAGCGCTTCCGACTTTCCT |  |
| GAPDH-F | CTGGAGAAACCTGCCAAGTATG | 138 |
| GAPDH-R | GGTGGAAGAATGGGAGTTGCT |  |
| NF-кB P65-F | CAGATACCACTAAGACGCACCC | 227 |
| NF-кB P65-R | CTCCAGGTCTCGCTTCTTCACA |  |
| Caspase 9-F | GACCAATGGGACTCAAATCAAAG | 208 |
| Caspase 9-R | TCGATCTTCCCTGGAGTACAGAC |  |
| Cytochrome c -F | GGTATCACCTGGGGAGAGGAT | 275 |
| Cytochrome c -R | TAAATCGGGGCTGTCCAACAA |  |
| CDK 4-F | TATGTGGAGTGTTGGCTGTATCTTC | 320 |
| CDK 4-R | CCGGGTCACTTTCCTCCTTGT |  |
| Caspase 3-F | CTGGACTGCGGTATTGAGACA | 103 |
| Caspase 3-R | CGGGTGCGGTAGAGTAAGC |  |

**Supplementary Table S2.** Evaluation parameters of PLS-DA models in the positive and negative ion mode.

| Group | NEG | | | POS | | |
| --- | --- | --- | --- | --- | --- | --- |
|  | *R^2^X*(cum) | *R^2^Y*(cum) | *Q^2^*(cum) | *R^2^X*(cum) | *R^2^Y*(cum) | *Q^2^*(cum) |
| MC vs NC | 0.774 | 0.997 | 0.828 | 0.754 | 0.991 | 0.791 |
| BDP-Ⅰ vs NC | 0.545 | 0.98 | 0.693 | 0.727 | 0.994 | 0.943 |
| BDP-Ⅰ vs MC | 0.491 | 0.88 | 0.566 | 0.673 | 0.703 | 0.4 |


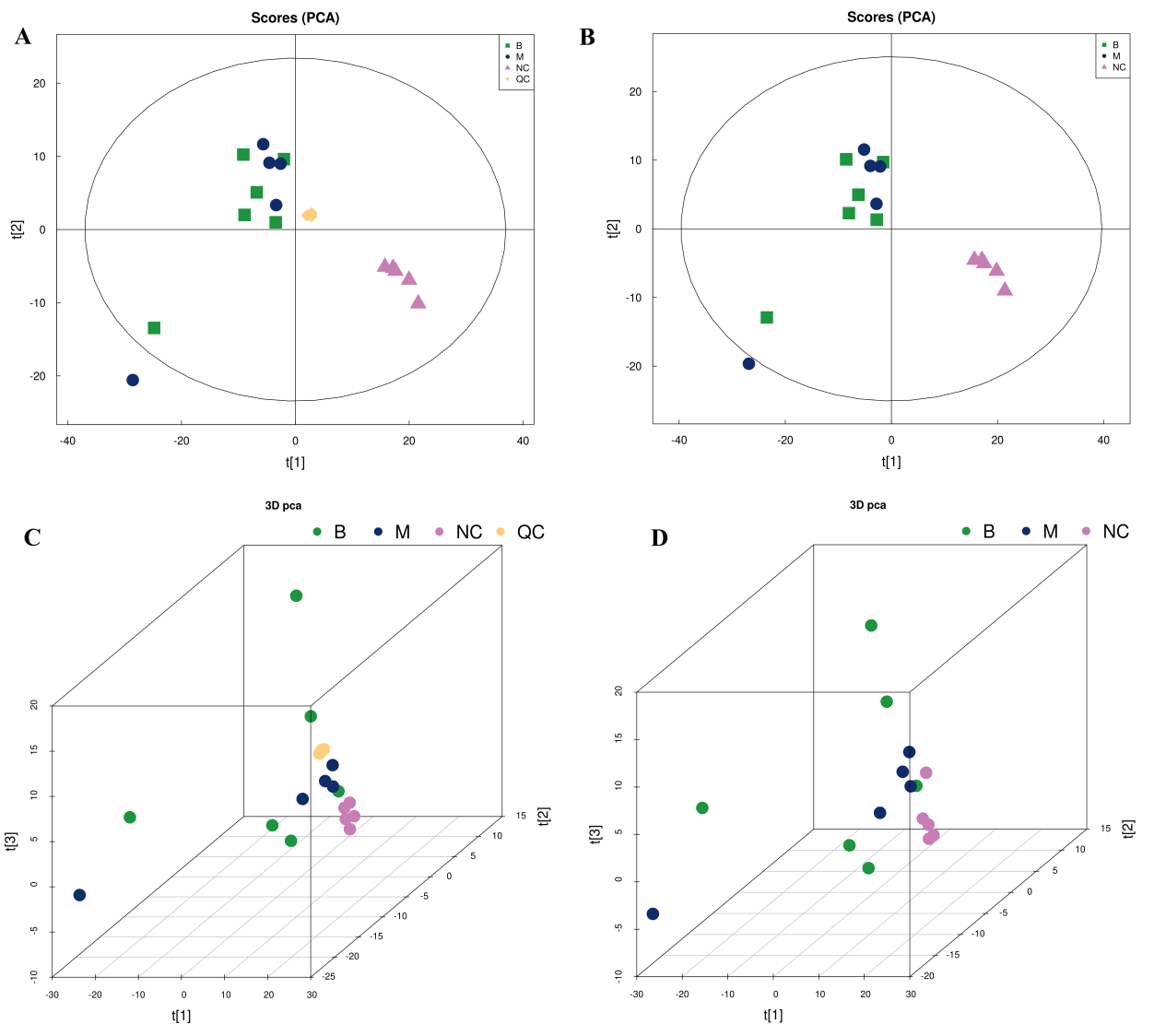


**Supplementary Figures S1** Quality control of meat samples. A and C. PCA analysis of all the samples based on peaks detected in positive ion modes. B and D. PCA analysis of all the samples based on peaks detected in negative ion modes.


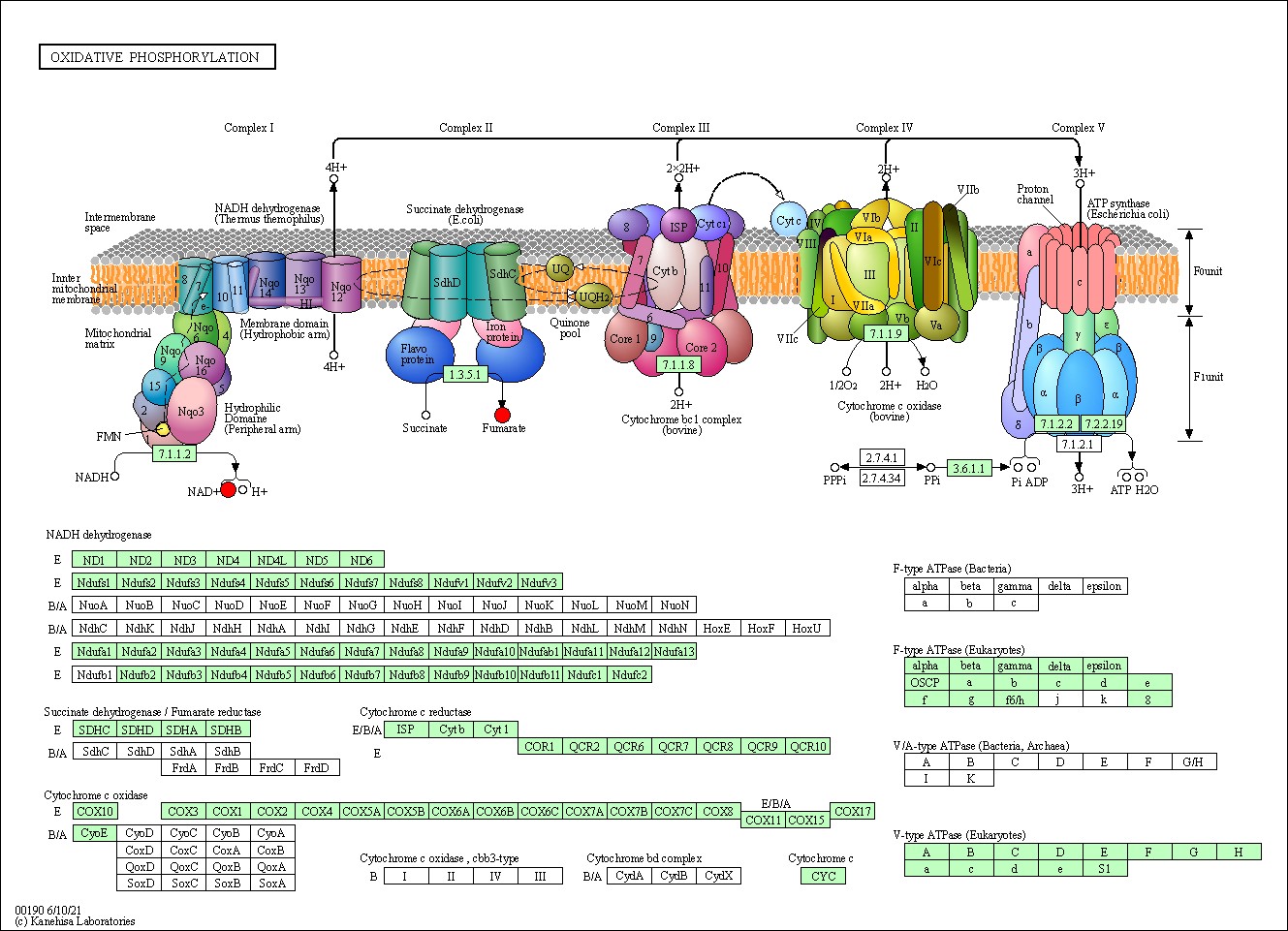


**Supplementary Figures S2** KEGG Pathway - Oxidative Phosphorylation Pathway
